# Use of *Agrobacterium rhizogenes* Strain 18r12v and Paromomycin Selection for Transformation of *Brachypodium distachyon* and *Brachypodium sylvaticum*

**DOI:** 10.3389/fpls.2016.00716

**Published:** 2016-05-24

**Authors:** Ray Collier, Jennifer Bragg, Bryan T. Hernandez, John P. Vogel, Roger Thilmony

**Affiliations:** ^1^Crop Improvement and Genetics Research Unit, Western Regional Research Center, United States Department of Agriculture-Agricultural Research Service, AlbanyCA, USA; ^2^Department of Plant Sciences, University of California, Davis, DavisCA, USA; ^3^Department of Energy, Joint Genome Institute, Walnut CreekCA, USA

**Keywords:** transformation, *Brachypodium*, *Agrobacterium rhizogenes*, paromomycin, tissue culture, callus

## Abstract

The genetic transformation of monocot grasses is a resource intensive process, the quality and efficiency of which is dependent in part upon the method of DNA introduction, as well as the ability to effectively separate transformed from wildtype tissue. *Agrobacterium*-mediated transformation of *Brachypodium* has relied mainly on *Agrobacterium tumefaciens* strain AGL1. Currently the antibiotic hygromycin B has been the selective agent of choice for robust identification of transgenic calli in *Brachypodium distachyon* and *Brachypodium sylvaticum* but few other chemicals have been shown to work as well for selection of transgenic *Brachypodium* cells in tissue culture. This study demonstrates that *Agrobacterium rhizogenes* strain 18r12v and paromomycin selection can be successfully used for the efficient generation of transgenic *B. distachyon* and *B. sylvaticum*. Additionally we observed that the transformation rates were similar to or higher than those obtained with *A*. *tumefaciens* strain AGL1 and hygromycin selection. The *A*. *rhizogenes* strain 18r12v harboring the pARS1 binary vector and paromomycin selection is an effective means of generating transgenic *Brachypodium* plants. This novel approach will facilitate the transgenic complementation of T-DNA knockout mutants of *B. distachyon* which were created using hygromycin selection, as well as aid the implementation of more complex genome manipulation strategies which require multiple rounds of transformation.

## Introduction

Genetic transformation of grasses is a technically challenging, labor and resource intensive endeavor which, unlike *Arabidopsis*, is dependent on tissue culture for the generation, propagation and maintenance of material for transformation experiments. Although a great deal of effort has been invested in streamlining protocols, significant bottlenecks remain that restrict the effective execution of complex biotechnological strategies geared toward metabolic pathway engineering, or recombinase mediated cassette exchange. The ability to efficiently select transgenic tissue/plants in tissue culture from a larger population of wildtype (WT) or previously modified tissue is one of the current limitations of grass transformation. A number of antibiotics and herbicides have been previously utilized for selection of transgenic monocot tissue/plants (Wilmink and Dons, 1993; Miki and McHugh, 2004; Sundar and Sakthivel, 2008; Rosellini, 2012; Bragg et al., 2015), but some of these compounds are not effective in tissue culture media and/or have been primarily used only as foliar applications. For the selection of transgenic callus of grasses, like the annual *Brachypodium distachyon* and the perennial *Brachypodium sylvaticum*, hygromycin B is the preferred compound (Hauptmann et al., 1988; Vain et al., 2008; Vogel and Hill, 2008; Thole and Vain, 2012; Steinwand et al., 2013), with resistance against this aminoglycoside antibiotic conferred to transgenic plants by expression of *APH(4′)*, also known as the *hygromycin phosphotransferase II* (*hptII*) gene from *Escherichia coli* (Waldron et al., 1985).

Other antibiotics have been assessed for their effectiveness in the selection of monocotyledonous tissue with limited success. Our previous studies directly compared Basta selection with hygromycin selection for the production of *B. distachyon* transgenic plants (Bragg et al., 2012). Regeneration efficiencies using Basta selection ranged from 5 to 23% of those achieved using hygromycin selection, and the survival of Basta transgenic plants through rooting to produce T_1_ seed was only half of that observed for hygromycin resistant transgenic plants. Notably, the antibiotic kanamycin, which has been used effectively for the selection of transgenic dicotyledonous plants, is not suitable for the grasses because of a high degree of natural tolerance to this compound (Hauptmann et al., 1988). Interestingly, NPTII, the enzyme frequently utilized to confer kanamycin resistance in dicot plants, can also detoxify other substrates including G418, neomycin, and paromomycin (Bevan et al., 1983; Guerche et al., 1987). Since paromomycin selection has been successfully used in an array of grasses (Torbert et al., 1995; Altpeter and Xu, 2000; Patnaik and Khurana, 2003; Popelka and Altpeter, 2003; Howe et al., 2006; Prakash et al., 2008; Long et al., 2011), and preliminary studies indicated that paromomycin could be used for selection in *B. distachyon* (Bragg et al., 2015; Rancour et al., 2015), we investigated conditions to optimize a robust, high-efficiency paromomycin selection scheme in *B. distachyon* and to test whether paromomycin selection could also be employed for *B. sylvaticum* transformation. Additionally, SHA17, an independently generated disarmed variant of *Agrobacterium rhizogenes* strain NCPPB2659, has previously been used to transform *Zea mays* (Mankin et al., 2007), thus we further explored the range of monocots which can be effectively transformed by 18r12v, another disarmed derivative of *A*. *rhizogenes* strain NCPPB2659 (Veena and Taylor, 2007).

## Materials and Methods

### Plasmid Construction and Transformation of *Agrobacterium* Strains

The pARS1 binary vector (GenBank KT985051) was constructed by first assembling a *Maize Ubiquitin1* (*ZmUbi1*) promoter-*nptII-CaMV 35S* terminator expression cassette. This was done by inserting an *nptII-35S* terminator fragment (amplified from pCAMBIA 2300 with the NptII_For 5′ aaaggatccACCATGGGGATTGAACAAGATG 3′ and NptII_Rev 5′ aaaggtacccgtacgCGTTTTTAATGTACTGAATTAACGCC 3′ primers) into the BamHI and KpnI sites of the pUbi-BASK plasmid. The pUbi-BASK plasmid (courtesy of James Thomson) is a modified pAHC20 vector (Christensen and Quail, 1996) which had the EcoRI restriction site within *ZmUbi1* promoter 5′ intron removed using site directed mutagenesis. The *bar* gene was excised from pAHC20 with BamHI and KpnI and two annealed oligos containing BamHI/AscI/SpeI/KpnI (BASK) restriction sites were inserted in its place between the *ZmUbi1* promoter and the *nos* terminator. The *ZmUbi1*p-*nptII-35S*t cassette was sequence confirmed and then excised with HindIII (blunted) and BsiWI and ligated into the pORE O3 binary vector (Coutu et al., 2007) digested with NcoI (blunted) and BsiWI.

**FIGURE 1:**
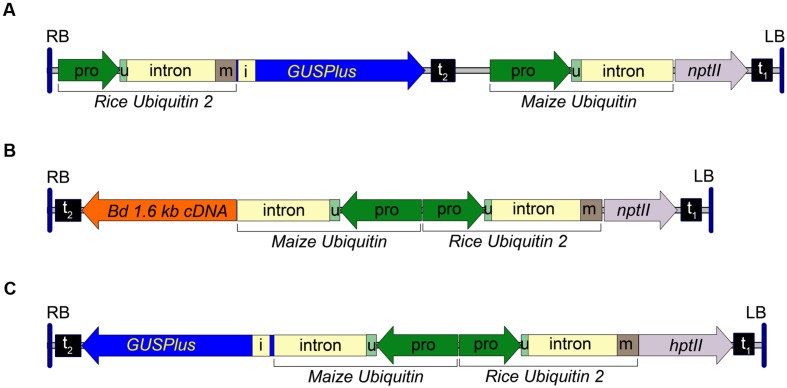
**Transfer DNA (T-DNA) region of the pARS1-RUBQ2-GUSPlus, pARS2-ZmUbi1-1.6 kb and pARS3m-ZmUbi1-GUSPlus binary vectors.** **(A)** The pARS1-RUBQ2-GUSPlus 8004 bp T-DNA with the synthetic *GUSPlus* reporter gene (Broothaerts et al., 2005) under control of the *Rice Ubiquitin*2 (*RUBQ2*) promoter (pro) – intron – monomer (m) (Wang et al., 2000) and terminated with the *nopaline synthase* (*nos*) terminator (t_2_) is shown. The coding sequence for the *RUBQ2* ubiquitin monomer (m) is translationally fused to *GUSPlus*. The *neomycin phosphotransferase* II (*nptII*) paromomycin resistance marker gene is under control of the *Maize Ubiquitin* 1 (*ZmUbi1*) promoter and intron (Christensen and Quail, 1996) with polyadenylation directed by the *Cauliflower Mosaic Virus* (*CaMV*) *35S* terminator (t_1_). **(B)** The pARS2-ZmUbi1-1.6 kb 8200 bp T-DNA with a *B. distachyon* cDNA under control of the *ZmUbi1* promoter and intron and terminated with the *nos* terminator (t_2_) is shown. The *RUBQ2* ubiquitin monomer (m) is translationally fused to the *nptII* paromomycin resistance marker gene and terminated by the *CaMV 35S* terminator (t_1_). **(C)** pARS3m-ZmUbi1-GUSPlus vector contains a 7757 bp T-DNA of with the *GUSPlus* gene under control of the *ZmUbi1* promoter and intron and the *nos* terminator (t_2_). The *RUBQ2* promoter – intron – monomer is translationally fused to the *hygromycin phosphotransferase* II (*hptII*) resistance gene and terminated with the *CaMV 35S* terminator (t_1_). The 5′ UTR (u) of each promoter is indicated. Each T-DNA is delineated by the right (RB) and left (LB) borders from the Ti plasmid of *A. tumefaciens* strain C58. Intron 1 (i) of the *Ricinus communis Catalase* 1 gene (Suzuki et al., 1994) in *GUSPlus* ensures that any plant tissue that exhibits β-glucuronidase activity is due to eukaryotic (spliced) expression of *GUSPlus*.

A rice ubiquitin 2 (*RUBQ2)* promoter/ubiquitin monomer-*GUSPlus-nos* terminator expression cassette was constructed by subcloning the *RUBQ2* promoter and 5′ intron from pGPro3 (GenBank JN593323) as a HindIII/KpnI fragment into pUbi-BASK. A 474bp portion of the *RUBQ2* intron was excised using PacI and PstI and replaced with a 739 bp synthetic fragment of DNA replacing the intron and adding the coding sequence for a single ubiquitin monomer. The resulting plasmid was digested with NcoI and MauBI and a 2354 bp *GUSPlus-nos* terminator fragment, excised from pCAMBIA 1305 with NcoI and MauBI, was inserted. The entire *RUBQ2*p-*GUSPlus-nos*t fragment was then sequence confirmed and excised with EcoRI and HindIII and inserted into the similarly digested pARS1 plasmid to form the pARS1-RUBQ2-GUSPlus binary vector. The T-DNA for the pARS1-RUBQ2-GUSPlus vector is shown in **Figure 1A** and the entire plasmid sequence is in **Supplemental File S1**.

To assemble the pARS1- 6kb and pARS1-7kb binary vectors, either a 6 kb *B. distachyon* genomic sequence (Bd3:36644481..36650622) flanked by KpnI sites or a 7 kb *B. distachyon* genomic sequence (Bd3:36644481..36651630) flanked by XbaI and KpnI sites was introduced into pARS1 digested with the same restriction enzymes. *B. distachyon* genomic sequences were PCR amplified from accession Bd21-3, and the amplified DNA was cloned into the pCR-BluntII-TOPO vector (Invitrogen, K2800-02) before transfer to pARS1.

The pARS2 binary vector (GenBank KT985052) was constructed from the pARS1-RUBQ2-GUSPlus vector by digesting with NcoI, excising the *GUSPlus-nos* terminator Maize *Ubi1* promoter fragment, and the re-ligating the plasmid. The multiple cloning site of this plasmid was then augmented by digesting with HindIII and PmeI and inserting a pORE O3 multiple cloning site fragments excised with MluI (Klenow blunted) and HindIII. A pARS2-ZmUbi1-GUSPlus binary vector was assembled by inserting a EcoRI-HindIII ZmUbi1-GUSPlus-nosT cassette (derived from a pUbi-BASK derived plasmid carrying the *GUSPlus* gene). The pARS2-ZmUbi1-1.6 kb binary vector was assembled by inserting an EcoRI-HindIII ZmUbi1-*B. distachyon* cDNA-nosT cassette (derived from a pUbi-BASK derived plasmid carrying a 1,614 bp Bradi1g54940 cDNA sequence) into the corresponding sites of pARS2. A diagram of the pARS2-ZmUbi1-1.6 kb T-DNA is shown in **Figure 1B** and the entire plasmid sequence for the pARS2-ZmUbi1-GUSPlus vector is in **Supplemental File S1**.

The pARS3m binary vector (GenBank KT985054) was assembled by the insertion of a *RUBQ2* promoter + monomer-*hptII-nos* terminator selection marker cassette into the pORE O3 binary vector similar to the strategy described above for pARS1. The *hptII* gene was derived from the pGPro8 binary vector (Genbank JN593327; Cook and Thilmony, 2012) and the multiple cloning site was augmented by the insertion of annealed oligos containing PmeI, MauBI, AscI and SbfI sites into the KpnI site. The pARS3m-ZmUbi-GUSPlus plasmid was constructed by insertion of a *ZmUbi1* promoter-*GUSPlus-nos* terminator expression cassette. The T-DNA for the pARS3m-ZmUbi1-GUSPlus vector is shown in **Figure 1C** and the entire plasmid sequence is in **Supplemental File S1**. The transformation vectors, bacterial strains and *Brachypodium* genotypes used in this research are available upon request.

### Transformation of *B. distachyon* and *B. sylvaticum*

*Brachypodium distachyon* Bd21-3 transformations with hygromycin selection were performed essentially as described (Bragg et al., 2012, 2015). Transformation of *B*. *distachyon* using paromomycin selection employed a similar protocol, but with the following modifications: Solid callus initiation media (CIM), solid regeneration media (RM), and MS/sucrose media used for rooting that contain 200–400 mg L^-1^ paromomycin sulfate (Phytotechnology #P710) and 150 mg L^-1^ timentin (RPI corp, Mount Prospect, IL #T36000) were prepared using 5 g L^-1^ Phyto agar (RPI Corp #A20300) as the gelling agent. Phyto agar (CAS Number 9002-18-0) was used as the gelling agent for the paromomycin containing media, instead of Phytagel (CAS Number 71010-52-1), following a previous report that paromomycin precipitates from media containing Phytagel (Kapaun and Cheng, 1999; Bragg et al., 2015). CIM per L: 4.43 g Linsmaier and Skoog (LS) basal medium (Phytotechnology, Shawnee Mission, Kansas #L689) (this may also be termed Murashige and Skoog minimal organics, MSMO), 30 g sucrose, 1 ml 0.6 mg ml^-1^ CuSO_4_, and 0.5 ml of 5 mg ml^-1^ 2,4-dichlorophenoxyacetic acid (Sigma–Aldrich) stock solution. Adjust media to pH 5.8 with 0.1 N KOH and sterilize by autoclaving. RM per L: 4.43 g LS basal medium, 30 g maltose, and 1.0 ml of 0.2 mg/ml kinetin stock solution. Adjust to pH 5.8 with 0.1 N KOH and sterilize by autoclaving. MS media per L: 4.42 g Murashige and Skoog (MS) basal medium with vitamins (Phytotechnology M519) and 30 g sucrose. Adjust to pH 5.7 with 0.1 N KOH and sterilize by autoclaving. The level of paromomycin sulfate used in the above media ranged from 200 to 400 mg L^-1^ as indicated in the text.

Transformation of *B*. *sylvaticum* followed the protocol of Steinwand et al. (2013), except that *Agrobacterium* strains were cultured on LB for single colonies for subsequent inoculation of liquid LB cultures for transformation. *Agrobacterium* cells were cultured for 24 h at 30°C and 270 rpm on an orbital shaker, and then collected by centrifugation for 10 min at 3000 *g* at 25°C in a swinging bucket rotor. The pellets were washed 1x with a volume of sterile Milli-Q water equal to the starting culture, then again pelleted by centrifugation. The supernatant was carefully removed and the pellet resuspended in inoculation medium (Steinwand et al., 2013) to an OD_600_ = 0.55, then used for a co-cultivation with *B*. *sylvaticum* callus for 10 min. The inoculation medium was removed by aspiration and the callus was then subjected to 3 days of desiccation (Steinwand et al., 2013). Subsequent to desiccation the tissue was cultured on selection, regeneration, and rooting media prepared with Phyto agar and containing paromomycin at 500 mg L^-1^.

### GUS Staining

Leaf, callus or seedling tissues were collected for β-glucuronidase activity detection. The GUS staining solution [0.1 M sodium phosphate pH 7.0, 0.5 mM potassium ferrocyanide, 0.5 mM potassium ferricyanide, 0.5% (v/v) Triton X-100, 0.15% (w/v) 5-Bromo-4-chloro-1H-indol-3-yl β-D-glucopyranosiduronic acid (X-gluc)] was added directly to the tubes and samples were vacuum infiltrated for 5 min before placing at 37°C in the dark overnight. The GUS staining solution was removed with a pipette, and 95% EtOH was added to remove any chlorophyll that might mask the blue staining and to fix the tissue.

### Genotyping of the Transgenic Plants

For *B*. *distachyon*, leaves were sampled directly into dilution buffer supplied with the Phire Plant Direct PCR kit (ThermoFisher F-130WH). Following manufacturer instructions, PCR amplification of the *nptII* gene from plant genomic DNA was performed with primers designed to yield a 700 bp product (NPTII F57 5′ GATTGAACAA GATGGATTGCACGC 3′ and NPTII R58 5′ CCACAG TCGATGAATCCAGAAAAGC 3′).

To confirm that paromomycin selected *B*. *sylvaticum* plants were transgenic approximately 10 mg of leaf sample was homogenized with 400 μl extraction buffer [200 mM Tris HCl, pH 7.5, 250 mM NaCl, 25 mM EDTA, 0.5% (w/v) SDS]. The cellular debris was pelleted by centrifugation and the supernatant was transferred. Genomic DNA was precipitated with isopropanol, and the resulting pellet was washed with 70% EtOH, dried, and then resuspended in sterile Milli-Q water. An aliquot of DNA from each independent line was used to confirm transgene integration in the plants by PCR amplification of an 870 bp product from the 3′ region of the *GUSPlus* gene with the primers GUSPlusF2Probe (5′ TTAACGAAGCGAGCAATGTG 3′) and GUSPlusR2Probe (5′ AGCCGAAATCTGGAATGTTG 3′).

### Seed Germination Test

Wild type Bd21-3 and transgenic T_1_ seeds were surface sterilized in a solution of 10% bleach and 0.4% Triton X-100 for 4 min shaking at room temperature. Surface sterilized seeds were washed three times with sterile water before placement on MS/sucrose plates containing 400 mg L^-1^ paromomycin sulfate, 150 mg L^-1^ timentin, and 5 g L^-1^ Phyto agar. Sealed plates were stored in a growth chamber at 28°C with a 20 h light/4 h dark cycle for germination.

## Results and Discussion

### Transformation of *B. distachyon* Using Paromomycin Selection

To investigate transformation of both *B. distachyon* and *B. sylvaticum* using *A. rhizogenes* strain 18r12v and paromomycin selection we constructed a novel series of binary vectors called pARS (see Materials and Methods). The pARS1 binary vector carries a T-DNA with a *Maize Ubiquitin1* (*ZmUbi1*) promoter (Christensen et al., 1992) – *nptII* selection marker expression cassette. An additional three pARS1 derivatives were also used in these studies. The pARS1-RUBQ2-GUSPlus binary vector contains a *GUSPlus* reporter gene whose expression is controlled by the *Rice Ubiquitin2* (*RUBQ2*) promoter (**Figure 1A**), and the pARS1-6 kb and pARS1-7 kb binary vectors include a cargo of either a 6 or 7 kb of *B. distachyon* genomic sequence. The pARS2 binary vector carries a T-DNA also with the *nptII* selection marker gene, but in this case, expression is controlled by the *RUBQ2* promoter and a ubiquitin monomer translational fusion (**Figure 1B**). The pARS2- ZmUbi1-1.6 kb derivative includes a *B. distachyon* cDNA cargo under control of the Maize *Ubiquitin1* promoter and intron (see Materials and Methods).

To optimize conditions for paromomycin sulfate selection of transgenic *B*. *distachyon* callus transformed with the *nptII* gene, CIM and RM media were prepared with 200, 300, or 400 mg L^-1^ paromomycin in place of the hygromycin B which is used for selection of callus transformed with the *hptII* gene (Bragg et al., 2012). Additionally, the Phytagel gelling agent (CAS Number 71010-52-1), used in CIM and RM plates with hygromycin B, was replaced with Phyto agar (CAS Number 9002-18-0), following a previous report that paromomycin precipitates from media containing Phytagel (Kapaun and Cheng, 1999).

**FIGURE 2:**
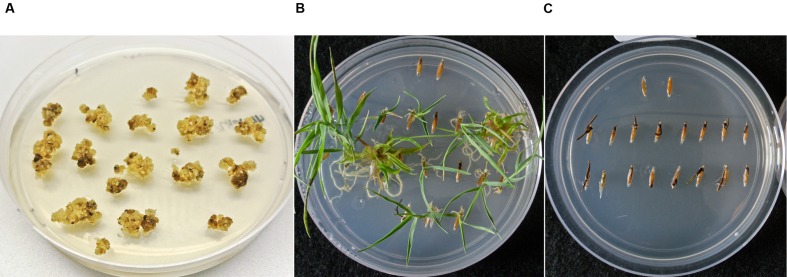
**Selection and segregation of *B. distachyon* on 400 mg L^-1^ paromomycin media.** **(A)** *B*. *distachyon* callus transformed with the pARS1-6 kb vector after 2 weeks under paromomycin selection. **(B)** Segregating transgenic T_1_ family with individuals exhibiting robust paromomycin resistance. **(C)** Non-transgenic line (escape) which is susceptible to paromomycin selection. For comparison purposes two wildtype (WT) seeds were included in the upper region of each germination plate. Plates were photographed 37 days after the seed was placed on the media.

After co-cultivation with AGL1 containing pARS1-6 kb, 68 pieces of Bd21-3 WT calli were transferred to CIM selective plates containing one of the three levels of paromomycin (200, 300, or 400 mg L^-1^). Pieces of callus recovered after co-cultivation with *Agrobacterium* were maintained separately through selection on CIM and RM. Multiple shoots representing families of siblings that regenerated from a single piece of callus were counted as a single transformation event. Transformation efficiency was calculated as the number of independent events recovered divided by the number of pieces of callus placed on selection. Over a period of 2 weeks, pieces of callus showed sectors of healthy, yellow growth with organized embryogenic structures surrounded by dark, necrotic tissue at all three antibiotic selection levels. Callus selected using 400 mg L^-1^ paromomycin showed the least growth (**Figure 2A**), whereas callus selected using the 200 and 300 mg L^-1^ levels continued to show relatively higher levels of tissue proliferation (data not shown). The resistant callus was then transferred to regeneration medium and any shoots that developed were transferred to non-selective medium for rooting. The 59 regenerants produced from this experiment were then screened for the presence of the *nptII* gene sequence using genomic PCR prior to transfer to soil. Overall, 20 of the 24 plants tested (83%) were positive for the *nptII* gene confirming they were transgenic and suggesting that the 4 remaining individuals were non-transgenic plants that survived paromomycin selection. Of the four identified non-transgenic escapes, two were selected on the 300 mg L^-1^ media and two were selected on the 400 mg L^-1^ media. The T_1_ seeds produced from 12 selfed T_0_ plants that tested PCR positive for the presence of the *nptII* gene were germinated on MS sucrose medium containing 400 mg L^-1^ paromomycin along with WT control seeds (**Figures 2B,C**; **Table 1**). The WT seeds failed to grow significantly on this medium, although the cotyledons sometimes emerged before growth halted. Nine of the twelve lines produced resistant T_1_ seedlings with strong growth on the selection medium (e.g., **Figure 2B**) and showed an overall segregation ratio consistent with expectations for a single transgenic locus based on chi square analyses (**Table 1**). These results confirm the heritability and functionality of the *nptII* selection marker transgene. Some of the T_1_ progeny from three other lines germinated but exhibited weak growth on selective medium (**Table 1**, marked with an asterisk). Though the growth of some of these T_1_ individuals was distinctly more than the WT controls, it was also significantly less than that observed for the progeny from other lines with robust paromomycin resistance. Seed germination for two of these families weakly resistant to paromomycin failed to conform to a 3:1 segregation ratio and likely contain transgenic individuals that lacked sufficient resistance to germinate and grow under this level of antibiotic selection. Progeny from three T_0_ regenerants that were identified by PCR as non-transgenic escapes failed to germinate under paromomycin selection and were indistinguishable from WT (**Figure 2C**). The results of this initial experiment demonstrated the utility of paromomycin selection and the functionality of the *ZmUbi1* promoter-*nptII* resistance marker in *B. distachyon*. Also evident from the results is that paromomycin selection performed during the callus and regeneration stages alone, allows some non-transgenic escape plants to survive.

**Table 1:** Segregation analysis of PCR confirmed paromomycin selected transgenic *B*. *distachyon* T_1_ families.

T0 Line Number	Total seeds	Paromomycin resistant	Paromomycin sensitive	Chi Square for 3:1	*P*-value
G8-10^∗^	15	3	12	24.20	<0.01
G8-13	24	15	9	2.00	0.16
G8-14	28	22	6	0.19	0.66
G8-16	34	23	11	0.98	0.32
G8-19	24	21	3	2.00	0.16
G8-20^∗^	22	9	13	13.64	<0.01
G8-21	16	14	2	1.33	0.25
G8-26	16	13	3	0.33	0.57
G8-28	14	12	2	0.86	0.35
G8-29^∗^	2	1	1	0.67	0.41
G8-31	19	16	3	0.86	0.35
G8-32	19	16	3	0.86	0.35


T_1_ seeds from paromomycin selected T_0_ plants were germinated on MS medium containing 400 mg L^-1^ paromomycin and seedling antibiotic resistance scored. *Lines with weak paromomycin resistance.

### Enhanced Selection of Transgenic *B. distachyon* Using Paromomycin in the Rooting Medium

AGL1 containing the pARS1-RUBQ2-GUSPlus vector, pARS1-6 kb, pARS1-7 kb, or pARS2-ZmUbi1-1.6 kb, were used for transformation of *B. distachyon* in three independent experiments. Calli for these experiments were derived from either WT Bd21-3 plants or from previously transformed *B. distachyon* Bd21-3 T-DNA lines that had been selected using hygromycin (24). For these experiments, transgenic calli were selected using 400 mg L^-1^ paromomycin maintained not only in the callus and RM, but also in the rooting medium. Each vector/callus combination was tested in two independent transformation experiments and the results are summarized in **Table 2**. A total of 1618 pieces of starting callus generated 964 (60% efficiency) green shoots, 119 (7%) albino shoots, and the remaining 535 (33%) pieces failed to regenerate any shoots after 6 weeks of selection. The source of the callus, generated from WT or previously transformed hygromycin resistant plants, did not substantially affect the transformation efficiency (62 and 57%). However, transformation efficiency for the vector containing the largest cargo (pARS1-7 kb) was reduced by 25–30% compared to the pARS1-RUBQ2-GUSPlus and pARS1-6 kb vectors in both experiments in which it was tested (**Table 2**).

**Table 2:** Transformation efficiency of *B*. *distachyon* selected on paromomycin.

Base vector	Cargo	Callus source	Callus pieces	Green events	Albino events	Efficiency (%)	^∗^nptII PCR +
pARS1	RUBQ2-GUSPlus	WT Bd21-3	238	169	17	71.0	^∗∗^42/43
pARS2	ZmUbi1-1.6kb	WT Bd21-3	504	292	72	57.9	33/34
				
				**Average Efficiency (%)**		**62.1**	

pARS1	RUBQ2-GUSPlus	HygR Bd21-3	64	40	0	62.5	^∗∗^N/A
pARS1	6kb	HygR Bd21-3	348	239	8	68.7	48/50
pARS1	7kb	HygR Bd21-3	464	224	22	48.3	48/49
				
				**Average Efficiency (%)**		**57.4**	

		**Overall sum**	**1618**	**964**	**119**		**171/176**
				
				**Average Efficiency (%)**		**59.6**	


*^∗^PCR positive plants/total plants tested. ^∗∗^This number includes plants generated using pARS1-RUBQ2-GUSPlus with both WT and hygromycin resistant Bd21-3 callus.*

A subset of the regenerating green shoots was transferred to rooting medium with paromomycin selection and only those individuals that produced substantial root growth with root hair formation were selected for transfer to soil. Leaf tissue from 176 regenerated and rooted plants was analyzed for the presence of the *nptII* gene sequence using genomic PCR (**Supplemental File S2**). The presence of the *nptII* sequence was confirmed for 171 (97%) of these plants. Five total escapes were identified, with only one or two escapes coming from each experiment (**Table 2**). These results demonstrate that paromomycin selection can be used for the efficient transformation of *B. distachyon* using vectors in which *nptII* is expressed by either the *ZmUbi1* or *RUBQ2* promoter as well as by vectors carrying a variety of cargos. Furthermore, paromomycin can be used to select for the introduction of additional DNA into previously transformed hygromycin resistant transgenic lines. Also it is clear that the likelihood of recovering escapes was not biased toward a particular vector or callus source and the recovery of non-transgenic escape plants can be minimized by the inclusion of paromomycin in the rooting medium.

### Transformation of *B. distachyon* with *A. rhizogenes* strain 18r12v

To evaluate the ability of *A. rhizogenes* strain 18r12v to transform *B. distachyon*, an experiment was conducted using either AGL1 or 18r12v carrying either the pARS1-RUBQ2-GUSPlus (**Figure 1A**) or the pARS3m-ZmUbi-GUSPlus (**Figure 1C**) binary vectors and employing paromomycin or hygromycin selection respectively. Embryogenic callus from *B*. *distachyon* was prepared and separated into four groups of approximately 200 pieces each for transformation with the different bacteria/plasmid combinations.

Highly efficient transformation was observed for all four combinations tested (**Table 3**): AGL1 with hygromycin selection (50%); AGL1 with paromomycin selection (43%); 18r12v with hygromycin selection (65%); and 18r12v with paromomycin selection (69%). These results show that the 18r12v transformations resulted in a higher efficiency than the AGL1 transformations performed in the paired experiment. The transformation efficiency of AGL1 using the two different selection schemes was similar (50 and 43%) as it was for the transformation conducted using 18r12v (65 and 69%). The number of calli producing only albino shoots was small and apparently not affected by either the strain or the selective agent used for transformation.

**Table 3:** Transformation efficiency of *B*. *distachyon* relative to *Agrobacterium* strain.

Strain	Selective agent	Calli (#)	Green events	Efficiency (%)	Albino	
	
					events	(%)
AGL1	Hygromycin	173	86	49.7	12	6.9
	Paromomycin	200	86	43.0	13	6.5
18r12v	Hygromycin	162	105	64.8	8	4.9
	Paromomycin	274	188	68.6	19	6.9


### *A. rhizogenes* Strain 18r12v-Mediated Transformation of *B. sylvaticum* Using Paromomycin Selection

**FIGURE 3:**
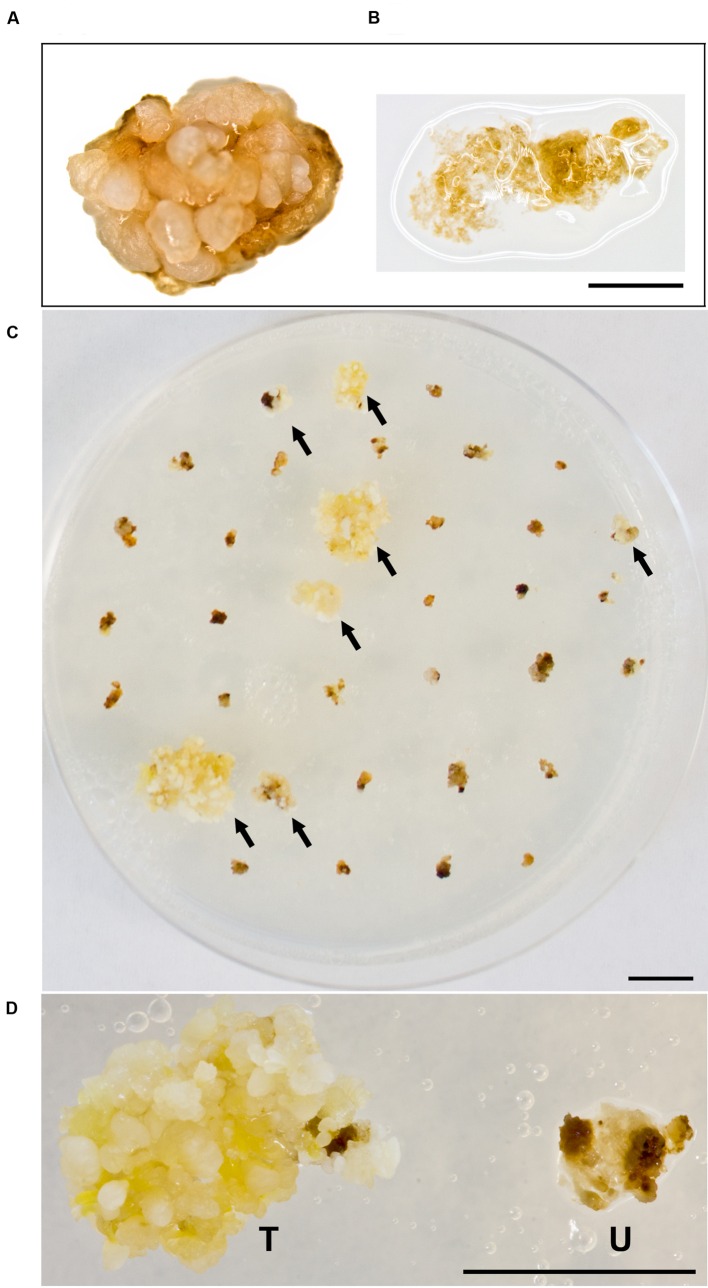
**Selection of *B. sylvaticum* callus on paromomycin media.** WT *B. sylvaticum* callus can tolerate paromomycin at 250 mg L^-1^ and grows **(A)** but at 750 mg L^-1^ **(B)** the tissue disintegrates with a watery appearance and dies; Scale bar = 1 mm. **(C)** *B*. *sylvaticum* tissue after 4 weeks under paromomycin selection at 500 mg L^-1^; arrows indicate transformed, paromomycin resistant calli; scale bar = 1 cm. **(D)** Magnified view of *B*. *sylvaticum* callus at 4 weeks under paromomycin selection; transformed callus (T) has a yellow-white appearance relative to untransformed callus (U) which is brown and dying; scale bar = 1 cm.

Building on what we learned about paromomycin selection of transgenic *B*. *distachyon*, we proceeded to adapt the protocol for use with *B*. *sylvaticum*. Embryogenic calli of *B*. *sylvaticum* line Ain-1, prepared as previously described (Steinwand et al., 2013), were transformed using 18r12v carrying the pARS1-RUBQ2-GUSPlus binary vector (**Figure 1A**). Since WT *B*. *sylvaticum* callus demonstrated tolerance to 250 mg L^-1^ paromomycin (**Figure 3A**), but not 750 mg L^-1^ (**Figure 3B**), a level of 500 mg L^-1^ paromomycin (**Figures 3C,D**) was utilized in all media through rooting to decrease the likelihood of recovering non-transgenic escapes. Two independent transformation experiments, which utilized separate batches of callus derived from embryos dissected on different days, were conducted to assess the efficacy of paromomycin selection as well as the suitability of 18r12v for *B*. *sylvaticum* transformation. Callus in the first experiment was split between 18r12v and AGL1 to assess the amount of paromomycin resistant regenerants produced by each strain. Independent transgenic events were assigned and the transformation efficiency was calculated following the method described previously for *B*. *distachyon*. Of the 284 pieces of callus co-cultivated with AGL1, 13 independent transgenic lines were recovered (4.6%), while from 379 calli co-cultured with 18r12v, 23 independent paromomycin resistant lines (multiple siblings for each) were generated (6.1%) (**Table 4**). From a second transformation experiment with 18r12v, an additional 11 independent paromomycin resistant lines were generated from 434 co-cultivated calli (**Table 4**). Leaf tissue from these T_0_ *B. sylvaticum* transgenic plants was analyzed for transgene activity by GUS staining, and genomic DNA was collected for further confirmation of the transgene presence by genomic PCR. The results (**Table 4**) show that no non-transformed escape plants were recovered from *B*. *sylvaticum* material selected with 500 mg L^-1^ paromomycin. The observed lower transformation efficiency (compared to *B. distachyon*) could be due to species differences and/or the higher level of selection that was used. It is noteworthy that this higher selection level allowed for no escapes, but may also be too stringent to recover weakly resistant transgenic regenerants.

**Table 4:** Paromomycin selected transgenic *B*. *sylvaticum*.

Experiment	Strain	Calli (#)	Events	GUS (+)		Efficiency (%)
					
				PCR	Stain	
1	AGL1	284	13	13/13	4/13	4.6
	18r12v	379	23	11/11	23/23	6.1
2	18r12v	434	11	11/11	7/11	2.5


Prior studies of the genetic transformation of *B. distachyon* and *B. sylvaticum* had a modest number of selective agents from which to choose. The availability of *A. rhizogenes* strain 18r12v and paromomycin selection will facilitate the efficient transformation of *Brachypodium* and can be particularly useful for the complementation of hygromycin resistant T-DNA insertion mutants. *Agrobacterium*-mediated plant transformation is a reliable and widely used technology, but there still remain opportunities for improvement and refinement of this important tool. Our results demonstrate that both *A*. *tumefaciens* strain AGL1 and *A*. *rhizogenes* strain 18r12v and hygromycin or paromomycin can be used to efficiently generate transgenic *Brachypodium* plants.

## Author Contributions

RC initiated the 18r12v experiments and performed the *B. sylvaticum* transformation, characterization and molecular analyses. JB initiated the paromomycin selection experiments, constructed the *B. distachyon* sequence containing vectors, performed the *B. distachyon* transformation and analyzed the resulting transgenic plants. BH assisted with the tissue culture and molecular characterization of the *B. sylvaticum* and *B. distachyon* plants. JV helped conceive of the research and supervised JB. RT conceived of the research, constructed the pARS vectors, supervised RC and BH and analyzed the results. All authors contributed to writing the manuscript and approved the final version.

## Conflict of Interest Statement

The authors declare that the research was conducted in the absence of any commercial or financial relationships that could be construed as a potential conflict of interest.
