# Supplementary material for: Use of Agrobacterium rhizogenes Strain 18r12v and Paromomycin Selection for Transformation of Brachypodium distachyon and Brachypodium sylvaticum
Source: Front Plant Sci. 2016 May 24;7:716. doi: 10.3389/fpls.2016.00716 (PMC4877385; doi:10.3389/fpls.2016.00716)
Supplement: Supplementary file 1 [file Data_Sheet_1.DOCX]

**Supplementary File 1**

**pARS1-RUBQ2-GUSPlus binary vector plasmid sequence**

**Annotation key: Right Border, RUBQ2 promoter, *RUBQ2 intron*, Ubiquitin monomer, GUSPlus, *catalase intron*, nos terminator (2x), Maize Ubi1 promoter, *Ubi1 intron*, nptII, 35S terminator, Left Border**

**GGTTTACCCGCCAATATATCCTGTCA**AACACTGATAGTTTAAACTGAAGGCGGGAAACGACAATCTGCTAGTGGATCTCCCAGTCACGACGTTGTAAAACGGGCGCCCCGCGGAAAGCTTATTCGGGTCAAGGCGGAAGCCAGCGCGCCACCCCACGTCAGCAAATACGGAGGCGCGGGGTTGACGGCGTCACCCGGTCCTAACGGCGACCAACAAACCAGCCAGAAGAAATTACAGTAAAAAAAAAGTAAATTGCACTTTGATCCACCTTTTATTACCTAAGTCTCAATTTGGATCACCCTTAAACCTATCTTTTCAATTTGGGCCGGGTTGTGGTTTGGACTACCATGAACAACTTTTCGTCATGTCTAACTTCCCTTTCAGCAAACATATGAACCATATATAGAGGAGATCGGCCGTATACTAGAGCTGATGTGTTTAAGGTCGTTGATTGCACGAGAAAAAAAAATCCAAATCGCAACAATAGCAAATTTATCTGGTTCAAAGTGAAAAGATATGTTTAAAGGTAGTCCAAAGTAAAACTTATAGATAATAAAATGTGGTCCAAAGCGTAATTCACTCAAAAAAAATCAACGAGACGTGTACCAAACGGAGACAAACGGCATCTTCTCGAAATTTCCCAACCGCTCGCTCGCCCGCCTCGTCTTCCCGGAAACCGCGGTGGTTTCAGCGTGGCGGATTCTCCAAGCAGACGGAGACGTCACGGCACGGGACTCCTCCCACCACCCAACCGCCATAAATACCAGCCCCCTCATCTCCTCTCCTCGCATCAGCTCCACCCCCGAAAAATTTCTCCCCAATCTCGCGAGGCTCTCGTCGTCGAATCGAATCCTCTCGCGTCCTCAAG*GTACGCTGCTTCTCCTCTCCTCGCTTCGTTTCGATTCGATTTCGGACGGGTGAGGTTGTTTTGTTGCTAGATCCGATTGGTGGTTAGGGTTGTCGATGTGATTATCGTGAGATGTTTAGGGGTTGTAGATCTGATGGTTGTGATTTGGGCACGGTTGGTTCGATAGGTGGAATCGTGGTTAGGTTTTGGGATTGGATGTTGGTTCTGATGATTGGGGGGAATTTTTACGGTTAGATGAATTGTTGGATGATTCGATTGGGGAAATCGGTGTAGATCTGTTGGGGAATTGTGGAACTAGTCATGCCTGAGTGATTGGTGCGATTTGTAGCGTGTTCCATCTTGTAGGCCTTGTTGCGAGCATGTTCAGATCTACTGTTCCGCTCTTGATTGAGTTATTGGTGCCTTGGGTTGGTGCAAACACAGGCTTTAATATGTTATATCTGTTTTGTGTTTGATGTAGATCTGTAGGGTAGTTCTTCTTAGACATGGTTCAATTATGTAGCTTGTGCGTTTCGATTTGATTTCATATGTTCACAGATTAGATAATGATGAACTCTTTTAATTAATTGTCAATGGTAAATAGGAAGTCTTGTCGCTATATCTGTCATAATGATCTCATGTTACTATCTGCCAGTAATTTATGCTAAGAACTATATTAGAATATCATGTTACAATCTGTAGTAATATCATGTTACAATCTGTAGTTCATCTATATAATCTATTGTGGTAATTTCTTTTTACTATCTGTGTGAAGATTATTGCCACTAGTTCATTCTACTTATTTCTGAAGTTCAGGATACGTGTGCTGTTACTACCTATCTGAATACATGTGTGATGTGCCTGTTACTATCTTTTTGAATACATGTATGTTCTGTTGGAATATGTTTGCTGTTTGATCCGTTGTTGTGTCCTTAATCTTGTGCTAGTTCTTACCCTATCTGTTTGGTGATTATTTCTTGCAG*ATGCAGATCTTTGTGAAGACATTGACCGGCAAGACTATCACCCTGGAGGTGGAGTCCTCTGACACCATCGACAATGTCAAGGCTAAGATCCAAGATAAGGAGGGCATCCCCCCGGACCAGCAGCGTCTCATCTTCGCTGGCAAGCAGCTCGAAGATGGCAGGACCCTTGCTGACTACAACATCCAGAAGGAGTCCACCCTTCACCTTGTCCTCCGCCTCCGTGGTGGCGGATCCATGGTAGATCTGAGG*GTAAATTTCTAGTTTTTCTCCTTCATTTTCTTGGTTAGGACCCTTTTCTCTTTTTATTTTTTTGAGCTTTGATCTTTCTTTAAACTGATCTATTTTTTAATTGATTGGTTATGGTGTAAATATTACATAGCTTTAACTGATAATCTGATTACTTTATTTCGTGTGTCTATGATGATGATGATAGTTACAG*AACCGACGAACTAGTCTGTACCCGATCAACACCGAGACCCGTGGCGTCTTCGACCTCAATGGCGTCTGGAACTTCAAGCTGGACTACGGGAAAGGACTGGAAGAGAAGTGGTACGAAAGCAAGCTGACCGACACTATTAGTATGGCCGTCCCAAGCAGTTACAATGACATTGGCGTGACCAAGGAAATCCGCAACCATATCGGATATGTCTGGTACGAACGTGAGTTCACGGTGCCGGCCTATCTGAAGGATCAGCGTATCGTGCTCCGCTTCGGCTCTGCAACTCACAAAGCAATTGTCTATGTCAATGGTGAGCTGGTCGTGGAGCACAAGGGCGGATTCCTGCCATTCGAAGCGGAAATCAACAACTCGCTGCGTGATGGCATGAATCGCGTCACCGTCGCCGTGGACAACATCCTCGACGATAGCACCCTCCCGGTGGGGCTGTACAGCGAGCGCCACGAAGAGGGCCTCGGAAAAGTCATTCGTAACAAGCCGAACTTCGACTTCTTCAACTATGCAGGCCTGCACCGTCCGGTGAAAATCTACACGACCCCGTTTACGTACGTCGAGGACATCTCGGTTGTGACCGACTTCAATGGCCCAACCGGGACTGTGACCTATACGGTGGACTTTCAAGGCAAAGCCGAGACCGTGAAAGTGTCGGTCGTGGATGAGGAAGGCAAAGTGGTCGCAAGCACCGAGGGCCTGAGCGGTAACGTGGAGATTCCGAATGTCATCCTCTGGGAACCACTGAACACGTATCTCTACCAGATCAAAGTGGAACTGGTGAACGACGGACTGACCATCGATGTCTATGAAGAGCCGTTCGGCGTGCGGACCGTGGAAGTCAACGACGGCAAGTTCCTCATCAACAACAAACCGTTCTACTTCAAGGGCTTTGGCAAACATGAGGACACTCCTATCAACGGCCGTGGCTTTAACGAAGCGAGCAATGTGATGGATTTCAATATCCTCAAATGGATCGGCGCCAACAGCTTCCGGACCGCACACTATCCGTACTCTGAAGAGTTGATGCGTCTTGCGGATCGCGAGGGTCTGGTCGTGATCGACGAGACTCCGGCAGTTGGCGTGCACCTCAACTTCATGGCCACCACGGGACTCGGCGAAGGCAGCGAGCGCGTCAGTACCTGGGAGAAGATTCGGACGTTTGAGCACCATCAAGACGTTCTCCGTGAACTGGTGTCTCGTGACAAGAACCATCCAAGCGTCGTGATGTGGAGCATCGCCAACGAGGCGGCGACTGAGGAAGAGGGCGCGTACGAGTACTTCAAGCCGTTGGTGGAGCTGACCAAGGAACTCGACCCACAGAAGCGTCCGGTCACGATCGTGCTGTTTGTGATGGCTACCCCGGAGACGGACAAAGTCGCCGAACTGATTGACGTCATCGCGCTCAATCGCTATAACGGATGGTACTTCGATGGCGGTGATCTCGAAGCGGCCAAAGTCCATCTCCGCCAGGAATTTCACGCGTGGAACAAGCGTTGCCCAGGAAAGCCGATCATGATCACTGAGTACGGCGCAGACACCGTTGCGGGCTTTCACGACATTGATCCAGTGATGTTCACCGAGGAATATCAAGTCGAGTACTACCAGGCGAACCACGTCGTGTTCGATGAGTTTGAGAACTTCGTGGGTGAGCAAGCGTGGAACTTCGCGGACTTCGCGACCTCTCAGGGCGTGATGCGCGTCCAAGGAAACAAGAAGGGCGTGTTCACTCGTGACCGCAAGCCGAAGCTCGCCGCGCACGTCTTTCGCGAGCGCTGGACCAACATTCCAGATTTCGGCTACAAGAACGCTAGCCATCACCATCACCATCACGTGTGACCTGCAGGTCTAGTGGTACCGCCCCGTCCGGTCCTGCCCGTCACCGAGATCTGATCCGTCGACCTGCAGATCGTTCAAACATTTGGCAATAAAGTTTCTTAAGATTGAATCCTGTTGCCGGTCTTGCGATGATTATCATATAATTTCTGTTGAATTACGTTAAGCATGTAATAATTAACATGTAATGCATGACGTTATTTATGAGATGGGTTTTTATGATTAGAGTCCCGCAATTATACATTTAATACGCGATAGAAAACAAAATATAGCGCGCAAACTAGGATAAATTATCGCGCGCGGTGTCATCTATGTTACTAGATCCGATGATAAGCTGTCAAACATGAGAATTCGTCGACTTTGCGGCCGCATCGATACTGCAGGAGCTCGGTACCTTTTACTAGTGATATCCCTGTGTGAAATTGTTATCCGCTACGCGTGATCGTTCAAACATTTGGCAATAAAGTTTCTTAAGATTGAATCCTGTTGCCGGTCTTGCGATGATTATCATATAATTTCTGTTGAATTACGTTAAGCATGTAATAATTAACATGTAATGCATGACGTTATTTATGAGATGGGTTTTTATGATTAGAGTCCCGCAATTATACATTTAATACGCGATAGAAAACAAAATATAGCGCGCAAACTAGGATAAATTATCGCGCGCGGTGTCATCTATGTTACTAGATCCCATGAGCTTGCATGCCTGCAGTGCAGCGTGACCCGGTCGTGCCCCTCTCTAGAGATAATGAGCATTGCATGTCTAAGTTATAAAAAATTACCACATATTTTTTTTGTCACACTTGTTTGAAGTGCAGTTTATCTATCTTTATACATATATTTAAACTTTACTCTACGAATAATATAATCTATAGTACTACAATAATATCAGTGTTTTAGAGAATCATATAAATGAACAGTTAGACATGGTCTAAAGGACAATTGAGTATTTTGACAACAGGACTCTACAGTTTTATCTTTTTAGTGTGCATGTGTTCTCCTTTTTTTTTGCAAATAGCTTCACCTATATAATACTTCATCCATTTTATTAGTACATCCATTTAGGGTTTAGGGTTAATGGTTTTTATAGACTAATTTTTTTAGTACATCTATTTTATTCTATTTTAGCCTCTAAATTAAGAAAACTAAAACTCTATTTTAGTTTTTTTATTTAATAATTTAGATATAAAATAGAATAAAATAAAGTGACTAAAAATTAAACAAATACCCTTTAAGAAATTAAAAAAACTAAGGAAACATTTTTCTTGTTTCGAGTAGATAATGCCAGCCTGTTAAACGCCGTCGACGAGTCTAACGGACACCAACCAGCGAACCAGCAGCGTCGCGTCGGGCCAAGCGAAGCAGACGGCACGGCATCTCTGTCGCTGCCTCTGGACCCCTCTCGAGAGTTCCGCTCCACCGTTGGACTTGCTCCGCTGTCGGCATCCAGAAATTGCGTGGCGGAGCGGCAGACGTGAGCCGGCACGGCAGGCGGCCTCCTCCTCCTCTCACGGCACCGGCAGCTACGGGGGATTCCTTTCCCACCGCTCCTTCGCTTTCCCTTCCTCGCCCGCCGTAATAAATAGACACCCCCTCCACACCCTCTTTCCCCAACCTCGTGTTGTTCGGAGCGCACACACACACAACCAGATCTCCCCCAAATCCACCCGTCGGCACCTCCGCTTCAAGGTACGCCGCTC*GTCCTCCCCCCCCCCCCCTCTCTACCTTCTCTAGATCGGCGTTCCGGTCCATGGTTAGGGCCCGGTAGTTCTACTTCTGTTCATGTTTGTGTTAGATCCGTGTTTGTGTTAGATCCGTGCTGCTAGCGTTCGTACACGGATGCGACCTGTACGTCAGACACGTTCTGATTGCTAACTTGCCAGTGTTTCTCTTTGGGGAATCCTGGGATGGCTCTAGCCGTTCCGCAGACGGGATCGATTTCATGATTTTTTTTGTTTCGTTGCATAGGGTTTGGTTTGCCCTTTTCCTTTATTTCAATATATGCCGTGCACTTGTTTGTCGGGTCATCTTTTCATGCTTTTTTTTGTCTTGGTTGTGATGATGTGGTCTGGTTGGGCGGTCGTTCTAGATCGGAGTAGAAATCTGTTTCAAACTACCTGGTGGATTTATTAATTTTGGATCTGTATGTGTGTGCCATACATATTCATAGTTACGAATTGAAGATGATGGATGGAAATATCGATCTAGGATAGGTATACATGTTGATGCGGGTTTTACTGATGCATATACAGAGATGCTTTTTGTTCGCTTGGTTGTGATGATGTGGTGTGGTTGGGCGGTCGTTCATTCGTTCTAGATCGGAGTAGAATACTGTTTCAAACTACCTGGTGTATTTATTAATTTTGGAACTGTATGTGTGTGTCATACATCTTCATAGTTACGAGTTTAAGATGGATGGAAATATCGATCTAGGATAGGTATACATGTTGATGTGGGTTTTACTGATGCATATACATGATGGCATATGCAGCATCTATTCATATGCTCTAACCTTGAGTACCTATCTATTATAATAAACAAGTATGTTTTATAATTATTTTGATCTTGATATACTTGGATGATGGCATATGCAGCAGCTATATGTGGATTTTTTTAGCCCTGCCTTCATACGCTATTTATTTGCTTGGTACTGTTTCTTTTGTCGATGCTCACCCTGTTGTTTGGTGTTACTTCTGCAG*GTCGACTCTAGAGGATCCGACCATGGGGATTGAACAAGATGGATTGCACGCAGGTTCTCCGGCCGCTTGGGTGGAGAGGCTATTCGGCTATGACTGGGCACAACAGACAATCGGCTGCTCTGATGCCGCCGTGTTCCGGCTGTCAGCGCAGGGGCGCCCGGTTCTTTTTGTCAAGACCGACCTGTCCGGTGCCCTGAATGAACTCCAGGACGAGGCAGCGCGGCTATCGTGGCTGGCCACGACGGGCGTTCCTTGCGCAGCTGTGCTCGACGTTGTCACTGAAGCGGGAAGGGACTGGCTGCTATTGGGCGAAGTGCCGGGGCAGGATCTCCTGTCATCTCACCTTGCTCCTGCCGAGAAAGTATCCATCATGGCTGATGCAATGCGGCGGCTGCATACGCTTGATCCGGCTACCTGCCCATTCGACCACCAAGCGAAACATCGCATCGAGCGAGCACGTACTCGGATGGAAGCCGGTCTTGTCGATCAGGATGATCTGGACGAAGAGCATCAGGGGCTCGCGCCAGCCGAACTGTTCGCCAGGCTCAAGGCGCGCATGCCCGACGGCGAGGATCTCGTCGTGACACATGGCGATGCCTGCTTGCCGAATATCATGGTGGAAAATGGCCGCTTTTCTGGATTCATCGACTGTGGCCGGCTGGGTGTGGCGGACCGCTATCAGGACATAGCGTTGGCTACCCGTGATATTGCTGAAGAGCTTGGCGGCGAATGGGCTGACCGCTTCCTCGTGCTTTACGGTATCGCCGCTCCCGATTCGCAGCGCATCGCCTTCTATCGCCTTCTTGACGAGTTCTTCTGAGCGGGACTCTGGGGTTCGGATCGATCCTCTAGCTAGAGTCGATCGACAAGCTCGAGTTTCTCCATAATAATGTGTGAGTAGTTCCCAGATAAGGGAATTAGGGTTCCTATAGGGTTTCGCTCATGTGTTGAGCATATAAGAAACCCTTAGTATGTATTTGTATTTGTAAAATACTTCTATCAATAAAATTTCTAATTCCTAAAACCAAAATCCAGTACTAAAATCCAGATCCCCCGAATTAATTCGGCGTTAATTCAGTACATTAAAAACGCGTACGGTTAAAACCACCCCAGTACATTAAAAACGTCCGCAATGTGTTATTAAGTTGTCTAAGCGTCAATTTGTTTACACCACAATATATCCTGCCACCAGCCAGCCAACAGCTCCCCGACCGGCAGCTCGGCACAAAATCACCACTCGATACAGGCAGCCCATCAGTCCACTAGACGCTCACCGGGCTGGTTGCCCTCGCCGCTGGGCTGGCGGCCGTCTATGGCCCTGCAAACGCGCCAGAAACGCCGTCGAAGCCGTGTGCGAGACACCGCAGCCGCCGGCGTTGTGGATACCTCGCGGAAAACTTGGCCCTCACTGACAGATGAGGGGCGGACGTTGACACTTGAGGGGCCGACTCACCCGGCGCGGCGTTGACAGATGAGGGGCAGGCTCGATTTCGGCCGGCGACGTGGAGCTGGCCAGCCTCGCAAATCGGCGAAAACGCCTGATTTTACGCGAGTTTCCCACAGATGATGTGGACAAGCCTGGGGATAAGTGCCCTGCGGTATTGACACTTGAGGGGCGCGACTACTGACAGATGAGGGGCGCGATCCTTGACACTTGAGGGGCAGAGTGCTGACAGATGAGGGGCGCACCTATTGACATTTGAGGGGCTGTCCACAGGCAGAAAATCCAGCATTTGCAAGGGTTTCCGCCCGTTTTTCGGCCACCGCTAACCTGTCTTTTAACCTGCTTTTAAACCAATATTTATAAACCTTGTTTTTAACCAGGGCTGCGCCCTGTGCGCGTGACCGCGCACGCCGAAGGGGGGTGCCCCCCCTTCTCGAACCCTCCCGGCCCGCTCTCGCGTTGGCAGCATCACCCATAATTGTGGTTTCAAAATCGGCTCCGTCGATACTATGTTATACGCCAACTTTGAAAACAACTTTGAAAAAGCTGTTTTCTGGTATTTAAGGTTTTAGAATGCAAGGAACAGTGAATTGGAGTTCGTCTTGTTATAATTAGCTTCTTGGGGTATCTTTAAATACTGTAGAAAAGAGGAAGGAAATAATAAATGGCTAAAATGAGAATATCACCGGAATTGAAAAAACTGATCGAAAAATACCGCTGCGTAAAAGATACGGAAGGAATGTCTCCTGCTAAGGTATATAAGCTGGTGGGAGAAAATGAAAACCTATATTTAAAAATGACGGACAGCCGGTATAAAGGGACCACCTATGATGTGGAACGGGAAAAGGACATGATGCTATGGCTGGAAGGAAAGCTGCCTGTTCCAAAGGTCCTGCACTTTGAACGGCATGATGGCTGGAGCAATCTGCTCATGAGTGAGGCCGATGGCGTCCTTTGCTCGGAAGAGTATGAAGATGAACAAAGCCCTGAAAAGATTATCGAGCTGTATGCGGAGTGCATCAGGCTCTTTCACTCCATCGACATATCGGATTGTCCCTATACGAATAGCTTAGACAGCCGCTTAGCCGAATTGGATTACTTACTGAATAACGATCTGGCCGATGTGGATTGCGAAAACTGGGAAGAAGACACTCCATTTAAAGATCCGCGCGAGCTGTATGATTTTTTAAAGACGGAAAAGCCCGAAGAGGAACTTGTCTTTTCCCACGGCGACCTGGGAGACAGCAACATCTTTGTGAAAGATGGCAAAGTAAGTGGCTTTATTGATCTTGGGAGAAGCGGCAGGGCGGACAAGTGGTATGACATTGCCTTCTGCGTCCGGTCGATCAGGGAGGATATTGGGGAAGAACAGTATGTCGAGCTATTTTTTGACTTACTGGGGATCAAGCCTGATTGGGAGAAAATAAAATATTATATTTTACTGGATGAATTGTTTTAGTACCTAGATGTGGCGCAACGATGCCGGCGACAAGCAGGAGCGCACCGACTTCTTCCGCATCAAGTGTTTTGGCTCTCAGGCCGAGGCCCACGGCAAGTATTTGGGCAAGGGGTCGCTGGTATTCGTGCAGGGCAAGATTCGGAATACCAAGTACGAGAAGGACGGCCAGACGGTCTACGGGACCGACTTCATTGCCGATAAGGTGGATTATCTGGACACCAAGGCACCAGGCGGGTCAAATCAGGAATAAGGGCACATTGCCCCGGCGTGAGTCGGGGCAATCCCGCAAGGAGGGTGAATGAATCGGACGTTTGACCGGAAGGCATACAGGCAAGAACTGATCGACGCGGGGTTTTCCGCCGAGGATGCCGAAACCATCGCAAGCCGCACCGTCATGCGTGCGCCCCGCGAAACCTTCCAGTCCGTCGGCTCGATGGTCCAGCAAGCTACGGCCAAGATCGAGCGCGACAGCGTGCAACTGGCTCCCCCTGCCCTGCCCGCGCCATCGGCCGCCGTGGAGCGTTCGCGTCGTCTCGAACAGGAGGCGGCAGGTTTGGCGAAGTCGATGACCATCGACACGCGAGGAACTATGACGACCAAGAAGCGAAAAACCGCCGGCGAGGACCTGGCAAAACAGGTCAGCGAGGCCAAGCAAGCCGCGTTGCTGAAACACACGAAGCAGCAGATCAAGGAAATGCAGCTTTCCTTGTTCGATATTGCGCCGTGGCCGGACACGATGCGAGCGATGCCAAACGACACGGCCCGCTCTGCCCTGTTCACCACGCGCAACAAGAAAATCCCGCGCGAGGCGCTGCAAAACAAGGTCATTTTCCACGTCAACAAGGACGTGAAGATCACCTACACCGGCGTCGAGCTGCGGGCCGACGATGACGAACTGGTGTGGCAGCAGGTGTTGGAGTACGCGAAGCGCACCCCTATCGGCGAGCCGATCACCTTCACGTTCTACGAGCTTTGCCAGGACCTGGGCTGGTCGATCAATGGCCGGTATTACACGAAGGCCGAGGAATGCCTGTCGCGCCTACAGGCGACGGCGATGGGCTTCACGTCCGACCGCGTTGGGCACCTGGAATCGGTGTCGCTGCTGCACCGCTTCCGCGTCCTGGACCGTGGCAAGAAAACGTCCCGTTGCCAGGTCCTGATCGACGAGGAAATCGTCGTGCTGTTTGCTGGCGACCACTACACGAAATTCATATGGGAGAAGTACCGCAAGCTGTCGCCGACGGCCCGACGGATGTTCGACTATTTCAGCTCGCACCGGGAGCCGTACCCGCTCAAGCTGGAAACCTTCCGCCTCATGTGCGGATCGGATTCCACCCGCGTGAAGAAGTGGCGCGAGCAGGTCGGCGAAGCCTGCGAAGAGTTGCGAGGCAGCGGCCTGGTGGAACACGCCTGGGTCAATGATGACCTGGTGCATTGCAAACGCTAGGGCCTTGTGGGGTCAGTTCCGGCTGGGGGTTCAGCAGCCAGCGCTTTACTGAGATCCTCTTCCGCTTCCTCGCTCACTGACTCGCTGCGCTCGGTCGTTCGGCTGCGGCGAGCGGTATCAGCTCACTCAAAGGCGGTAATACGGTTATCCACAGAATCAGGGGATAACGCAGGAAAGAACATGTGAGCAAAAGGCCAGCAAAAGGCCAGGAACCGTAAAAAGGCCGCGTTGCTGGCGTTTTTCCATAGGCTCCGCCCCCCTGACGAGCATCACAAAAATCGACGCTCAAGTCAGAGGTGGCGAAACCCGACAGGACTATAAAGATACCAGGCGTTTCCCCCTGGAAGCTCCCTCGTGCGCTCTCCTGTTCCGACCCTGCCGCTTACCGGATACCTGTCCGCCTTTCTCCCTTCGGGAAGCGTGGCGCTTTCTCATAGCTCACGCTGTAGGTATCTCAGTTCGGTGTAGGTCGTTCGCTCCAAGCTGGGCTGTGTGCACGAACCCCCCGTTCAGCCCGACCGCTGCGCCTTATCCGGTAACTATCGTCTTGAGTCCAACCCGGTAAGACACGACTTATCGCCACTGGCAGCAGCCACTGGTAACAGGATTAGCAGAGCGAGGTATGTAGGCGGTGCTACAGAGTTCTTGAAGTGGTGGCCTAACTACGGCTACACTAGAAGAACAGTATTTGGTATCTGCGCTCTGCTGAAGCCAGTTACCTTCGGAAAAAGAGTTGGTAGCTCTTGATCCGGCAAACAAACCACCGCTGGTAGCGGTGGTTTTTTTGTTTGCAAGCAGCAGATTACGCGCAGAAAAAAAGGATCTCAAGAAGATCCTTTGATCTTTTCTACGGGGTCTGACGCTCAGTGGAACGAAAACTCACGTTAAGGGATTTTGGTCATGAGATTATCAAAAAGGATCTTCACCTAGATCCTTTTGGATCTCCTGTGGTTGGCATGCACATACAAATGGACGAACGGATAAACCTTTTCACGCCCTTTTAAATATCCGATTATTCTAATAAACGCTCTTTTCTCTTA

**pARS2-ZmUbi1-GUSPlus binary vector plasmid sequence**

**Annotation key: Right Border, nos terminator, GUSPlus, *catalase intron*, *Ubi1 intron*, Maize Ubi1 promoter, RUBQ2 promoter, *RUBQ2 intron*, Ubiquitin monomer, nptII, 35S terminator, Left Border**

**GGTTTACCCGCCAATATATCCTGTCA**AACACTGATAGTTTCGCGTAGCGGATAACAATTTCACACAGGGATATCACTAGTAAAAGGTACCGAGCTCCTGCAGTATCGATGCGGCCGCAAAGTCGACGAATTCTCATGTTTGACAGCTTATCATCGGATCTAGTAACATAGATGACACCGCGCGCGATAATTTATCCTAGTTTGCGCGCTATATTTTGTTTTCTATCGCGTATTAAATGTATAATTGCGGGACTCTAATCATAAAAACCCATCTCATAAATAACGTCATGCATTACATGTTAATTATTACATGCTTAACGTAATTCAACAGAAATTATATGATAATCATCGCAAGACCGGCAACAGGATTCAATCTTAAGAAACTTTATTGCCAAATGTTTGAACGATCTGCAGGTCGACGGATCAGATCTCGGTGACGGGCAGGACCGGACGGGGCGGTACCACTAGACCTGCAGGTCACACGTGATGGTGATGGTGATGGCTAGCGTTCTTGTAGCCGAAATCTGGAATGTTGGTCCAGCGCTCGCGAAAGACGTGCGCGGCGAGCTTCGGCTTGCGGTCACGAGTGAACACGCCCTTCTTGTTTCCTTGGACGCGCATCACGCCCTGAGAGGTCGCGAAGTCCGCGAAGTTCCACGCTTGCTCACCCACGAAGTTCTCAAACTCATCGAACACGACGTGGTTCGCCTGGTAGTACTCGACTTGATATTCCTCGGTGAACATCACTGGATCAATGTCGTGAAAGCCCGCAACGGTGTCTGCGCCGTACTCAGTGATCATGATCGGCTTTCCTGGGCAACGCTTGTTCCACGCGTGAAATTCCTGGCGGAGATGGACTTTGGCCGCTTCGAGATCACCGCCATCGAAGTACCATCCGTTATAGCGATTGAGCGCGATGACGTCAATCAGTTCGGCGACTTTGTCCGTCTCCGGGGTAGCCATCACAAACAGCACGATCGTGACCGGACGCTTCTGTGGGTCGAGTTCCTTGGTCAGCTCCACCAACGGCTTGAAGTACTCGTACGCGCCCTCTTCCTCAGTCGCCGCCTCGTTGGCGATGCTCCACATCACGACGCTTGGATGGTTCTTGTCACGAGACACCAGTTCACGGAGAACGTCTTGATGGTGCTCAAACGTCCGAATCTTCTCCCAGGTACTGACGCGCTCGCTGCCTTCGCCGAGTCCCGTGGTGGCCATGAAGTTGAGGTGCACGCCAACTGCCGGAGTCTCGTCGATCACGACCAGACCCTCGCGATCCGCAAGACGCATCAACTCTTCAGAGTACGGATAGTGTGCGGTCCGGAAGCTGTTGGCGCCGATCCATTTGAGGATATTGAAATCCATCACATTGCTCGCTTCGTTAAAGCCACGGCCGTTGATAGGAGTGTCCTCATGTTTGCCAAAGCCCTTGAAGTAGAACGGTTTGTTGTTGATGAGGAACTTGCCGTCGTTGACTTCCACGGTCCGCACGCCGAACGGCTCTTCATAGACATCGATGGTCAGTCCGTCGTTCACCAGTTCCACTTTGATCTGGTAGAGATACGTGTTCAGTGGTTCCCAGAGGATGACATTCGGAATCTCCACGTTACCGCTCAGGCCCTCGGTGCTTGCGACCACTTTGCCTTCCTCATCCACGACCGACACTTTCACGGTCTCGGCTTTGCCTTGAAAGTCCACCGTATAGGTCACAGTCCCGGTTGGGCCATTGAAGTCGGTCACAACCGAGATGTCCTCGACGTACGTAAACGGGGTCGTGTAGATTTTCACCGGACGGTGCAGGCCTGCATAGTTGAAGAAGTCGAAGTTCGGCTTGTTACGAATGACTTTTCCGAGGCCCTCTTCGTGGCGCTCGCTGTACAGCCCCACCGGGAGGGTGCTATCGTCGAGGATGTTGTCCACGGCGACGGTGACGCGATTCATGCCATCACGCAGCGAGTTGTTGATTTCCGCTTCGAATGGCAGGAATCCGCCCTTGTGCTCCACGACCAGCTCACCATTGACATAGACAATTGCTTTGTGAGTTGCAGAGCCGAAGCGGAGCACGATACGCTGATCCTTCAGATAGGCCGGCACCGTGAACTCACGTTCGTACCAGACATATCCGATATGGTTGCGGATTTCCTTGGTCACGCCAATGTCATTGTAACTGCTTGGGACGGCCATACTAATAGTGTCGGTCAGCTTGCTTTCGTACCACTTCTCTTCCAGTCCTTTCCCGTAGTCCAGCTTGAAGTTCCAGACGCCATTGAGGTCGAAGACGCCACGGGTCTCGGTGTTGATCGGGTACAGACTAGTTCGTCGGTT*CTGTAACTATCATCATCATCATAGACACACGAAATAAAGTAATCAGATTATCAGTTAAAGCTATGTAATATTTACACCATAACCAATCAATTAAAAAATAGATCAGTTTAAAGAAAGATCAAAGCTCAAAAAAATAAAAAGAGAAAAGGGTCCTAACCAAGAAAATGAAGGAGAAAAACTAGAAATTTAC*CCTCAGATCTACCATGGTTGGCGCGCCGGATCCTCTAGAGTCGAC*CTGCAGAAGTAACACCAAACAACAGGGTGAGCATCGACAAAAGAAACAGTACCAAGCAAATAAATAGCGTATGAAGGCAGGGCTAAAAAAATCCACATATAGCTGCTGCATATGCCATCATCCAAGTATATCAAGATCAAAATAATTATAAAACATACTTGTTTATTATAATAGATAGGTACTCAAGGTTAGAGCATATGAATAGATGCTGCATATGCCATCATGTATATGCATCAGTAAAACCCACATCAACATGTATACCTATCCTAGATCGATATTTCCATCCATCTTAAACTCGTAACTATGAAGATGTATGACACACACATACAGTTCCAAAATTAATAAATACACCAGGTAGTTTGAAACAGTATTCTACTCCGATCTAGAACGAATGAACGACCGCCCAACCACACCACATCATCACAACCAAGCGAACAAAAAGCATCTCTGTATATGCATCAGTAAAACCCGCATCAACATGTATACCTATCCTAGATCGATATTTCCATCCATCATCTTCAATTCGTAACTATGAATATGTATGGCACACACATACAGATCCAAAATTAATAAATCCACCAGGTAGTTTGAAACAGATTTCTACTCCGATCTAGAACGACCGCCCAACCAGACCACATCATCACAACCAAGACAAAAAAAAGCATGAAAAGATGACCCGACAAACAAGTGCACGGCATATATTGAAATAAAGGAAAAGGGCAAACCAAACCCTATGCAACGAAACAAAAAAAATCATGAAATCGATCCCGTCTGCGGAACGGCTAGAGCCATCCCAGGATTCCCCAAAGAGAAACACTGGCAAGTTAGCAATCAGAACGTGTCTGACGTACAGGTCGCATCCGTGTACGAACGCTAGCAGCACGGATCTAACACAAACACGGATCTAACACAAACATGAACAGAAGTAGAACTACCGGGCCCTAACCATGGACCGGAACGCCGATCTAGAGAAGGTAGAGAGGGGGGGGGGGGGAGGAC*GAGCGGCGTACCTTGAAGCGGAGGTGCCGACGGGTGGATTTGGGGGAGATCTGGTTGTGTGTGTGTGCGCTCCGAACAACACGAGGTTGGGGAAAGAGGGTGTGGAGGGGGTGTCTATTTATTACGGCGGGCGAGGAAGGGAAAGCGAAGGAGCGGTGGGAAAGGAATCCCCCGTAGCTGCCGGTGCCGTGAGAGGAGGAGGAGGCCGCCTGCCGTGCCGGCTCACGTCTGCCGCTCCGCCACGCAATTTCTGGATGCCGACAGCGGAGCAAGTCCAACGGTGGAGCGGAACTCTCGAGAGGGGTCCAGAGGCAGCGACAGAGATGCCGTGCCGTCTGCTTCGCTTGGCCCGACGCGACGCTGCTGGTTCGCTGGTTGGTGTCCGTTAGACTCGTCGACGGCGTTTAACAGGCTGGCATTATCTACTCGAAACAAGAAAAATGTTTCCTTAGTTTTTTTAATTTCTTAAAGGGTATTTGTTTAATTTTTAGTCACTTTATTTTATTCTATTTTATATCTAAATTATTAAATAAAAAAACTAAAATAGAGTTTTAGTTTTCTTAATTTAGAGGCTAAAATAGAATAAAATAGATGTACTAAAAAAATTAGTCTATAAAAACCATTAACCCTAAACCCTAAATGGATGTACTAATAAAATGGATGAAGTATTATATAGGTGAAGCTATTTGCAAAAAAAAAGGAGAACACATGCACACTAAAAAGATAAAACTGTAGAGTCCTGTTGTCAAAATACTCAATTGTCCTTTAGACCATGTCTAACTGTTCATTTATATGATTCTCTAAAACACTGATATTATTGTAGTACTATAGATTATATTATTCGTAGAGTAAAGTTTAAATATATGTATAAAGATAGATAAACTGCACTTCAAACAAGTGTGACAAAAAAAATATGTGGTAATTTTTTATAACTTAGACATGCAATGCTCATTATCTCTAGAGAGGGGCACGACCGGGTCACGCTGCACTGCAGGCATGCAAGCTTATTCGGGTCAAGGCGGAAGCCAGCGCGCCACCCCACGTCAGCAAATACGGAGGCGCGGGGTTGACGGCGTCACCCGGTCCTAACGGCGACCAACAAACCAGCCAGAAGAAATTACAGTAAAAAAAAAGTAAATTGCACTTTGATCCACCTTTTATTACCTAAGTCTCAATTTGGATCACCCTTAAACCTATCTTTTCAATTTGGGCCGGGTTGTGGTTTGGACTACCATGAACAACTTTTCGTCATGTCTAACTTCCCTTTCAGCAAACATATGAACCATATATAGAGGAGATCGGCCGTATACTAGAGCTGATGTGTTTAAGGTCGTTGATTGCACGAGAAAAAAAAATCCAAATCGCAACAATAGCAAATTTATCTGGTTCAAAGTGAAAAGATATGTTTAAAGGTAGTCCAAAGTAAAACTTATAGATAATAAAATGTGGTCCAAAGCGTAATTCACTCAAAAAAAATCAACGAGACGTGTACCAAACGGAGACAAACGGCATCTTCTCGAAATTTCCCAACCGCTCGCTCGCCCGCCTCGTCTTCCCGGAAACCGCGGTGGTTTCAGCGTGGCGGATTCTCCAAGCAGACGGAGACGTCACGGCACGGGACTCCTCCCACCACCCAACCGCCATAAATACCAGCCCCCTCATCTCCTCTCCTCGCATCAGCTCCACCCCCGAAAAATTTCTCCCCAATCTCGCGAGGCTCTCGTCGTCGAATCGAATCCTCTCGCGTCCTCAAG*GTACGCTGCTTCTCCTCTCCTCGCTTCGTTTCGATTCGATTTCGGACGGGTGAGGTTGTTTTGTTGCTAGATCCGATTGGTGGTTAGGGTTGTCGATGTGATTATCGTGAGATGTTTAGGGGTTGTAGATCTGATGGTTGTGATTTGGGCACGGTTGGTTCGATAGGTGGAATCGTGGTTAGGTTTTGGGATTGGATGTTGGTTCTGATGATTGGGGGGAATTTTTACGGTTAGATGAATTGTTGGATGATTCGATTGGGGAAATCGGTGTAGATCTGTTGGGGAATTGTGGAACTAGTCATGCCTGAGTGATTGGTGCGATTTGTAGCGTGTTCCATCTTGTAGGCCTTGTTGCGAGCATGTTCAGATCTACTGTTCCGCTCTTGATTGAGTTATTGGTGCCTTGGGTTGGTGCAAACACAGGCTTTAATATGTTATATCTGTTTTGTGTTTGATGTAGATCTGTAGGGTAGTTCTTCTTAGACATGGTTCAATTATGTAGCTTGTGCGTTTCGATTTGATTTCATATGTTCACAGATTAGATAATGATGAACTCTTTTAATTAATTGTCAATGGTAAATAGGAAGTCTTGTCGCTATATCTGTCATAATGATCTCATGTTACTATCTGCCAGTAATTTATGCTAAGAACTATATTAGAATATCATGTTACAATCTGTAGTAATATCATGTTACAATCTGTAGTTCATCTATATAATCTATTGTGGTAATTTCTTTTTACTATCTGTGTGAAGATTATTGCCACTAGTTCATTCTACTTATTTCTGAAGTTCAGGATACGTGTGCTGTTACTACCTATCTGAATACATGTGTGATGTGCCTGTTACTATCTTTTTGAATACATGTATGTTCTGTTGGAATATGTTTGCTGTTTGATCCGTTGTTGTGTCCTTAATCTTGTGCTAGTTCTTACCCTATCTGTTTGGTGATTATTTCTTGCAG*ATGCAGATCTTTGTGAAGACATTGACCGGCAAGACTATCACCCTGGAGGTGGAGTCCTCTGACACCATCGACAATGTCAAGGCTAAGATCCAAGATAAGGAGGGCATCCCCCCGGACCAGCAGCGTCTCATCTTCGCTGGCAAGCAGCTCGAAGATGGCAGGACCCTTGCTGACTACAACATCCAGAAGGAGTCCACCCTTCACCTTGTCCTCCGCCTCCGTGGTGGCGGATCCATGGGGATTGAACAAGATGGATTGCACGCAGGTTCTCCGGCCGCTTGGGTGGAGAGGCTATTCGGCTATGACTGGGCACAACAGACAATCGGCTGCTCTGATGCCGCCGTGTTCCGGCTGTCAGCGCAGGGGCGCCCGGTTCTTTTTGTCAAGACCGACCTGTCCGGTGCCCTGAATGAACTCCAGGACGAGGCAGCGCGGCTATCGTGGCTGGCCACGACGGGCGTTCCTTGCGCAGCTGTGCTCGACGTTGTCACTGAAGCGGGAAGGGACTGGCTGCTATTGGGCGAAGTGCCGGGGCAGGATCTCCTGTCATCTCACCTTGCTCCTGCCGAGAAAGTATCCATCATGGCTGATGCAATGCGGCGGCTGCATACGCTTGATCCGGCTACCTGCCCATTCGACCACCAAGCGAAACATCGCATCGAGCGAGCACGTACTCGGATGGAAGCCGGTCTTGTCGATCAGGATGATCTGGACGAAGAGCATCAGGGGCTCGCGCCAGCCGAACTGTTCGCCAGGCTCAAGGCGCGCATGCCCGACGGCGAGGATCTCGTCGTGACACATGGCGATGCCTGCTTGCCGAATATCATGGTGGAAAATGGCCGCTTTTCTGGATTCATCGACTGTGGCCGGCTGGGTGTGGCGGACCGCTATCAGGACATAGCGTTGGCTACCCGTGATATTGCTGAAGAGCTTGGCGGCGAATGGGCTGACCGCTTCCTCGTGCTTTACGGTATCGCCGCTCCCGATTCGCAGCGCATCGCCTTCTATCGCCTTCTTGACGAGTTCTTCTGAGCGGGACTCTGGGGTTCGGATCGATCCTCTAGCTAGAGTCGATCGACAAGCTCGAGTTTCTCCATAATAATGTGTGAGTAGTTCCCAGATAAGGGAATTAGGGTTCCTATAGGGTTTCGCTCATGTGTTGAGCATATAAGAAACCCTTAGTATGTATTTGTATTTGTAAAATACTTCTATCAATAAAATTTCTAATTCCTAAAACCAAAATCCAGTACTAAAATCCAGATCCCCCGAATTAATTCGGCGTTAATTCAGTACATTAAAAACGCGTACGGTTAAAACCACCCCAGTACATTAAAAACGTCCGCAATGTGTTATTAAGTTGTCTAAGCGTCAATTTGTTTACACCACAATATATCCTGCCACCAGCCAGCCAACAGCTCCCCGACCGGCAGCTCGGCACAAAATCACCACTCGATACAGGCAGCCCATCAGTCCACTAGACGCTCACCGGGCTGGTTGCCCTCGCCGCTGGGCTGGCGGCCGTCTATGGCCCTGCAAACGCGCCAGAAACGCCGTCGAAGCCGTGTGCGAGACACCGCAGCCGCCGGCGTTGTGGATACCTCGCGGAAAACTTGGCCCTCACTGACAGATGAGGGGCGGACGTTGACACTTGAGGGGCCGACTCACCCGGCGCGGCGTTGACAGATGAGGGGCAGGCTCGATTTCGGCCGGCGACGTGGAGCTGGCCAGCCTCGCAAATCGGCGAAAACGCCTGATTTTACGCGAGTTTCCCACAGATGATGTGGACAAGCCTGGGGATAAGTGCCCTGCGGTATTGACACTTGAGGGGCGCGACTACTGACAGATGAGGGGCGCGATCCTTGACACTTGAGGGGCAGAGTGCTGACAGATGAGGGGCGCACCTATTGACATTTGAGGGGCTGTCCACAGGCAGAAAATCCAGCATTTGCAAGGGTTTCCGCCCGTTTTTCGGCCACCGCTAACCTGTCTTTTAACCTGCTTTTAAACCAATATTTATAAACCTTGTTTTTAACCAGGGCTGCGCCCTGTGCGCGTGACCGCGCACGCCGAAGGGGGGTGCCCCCCCTTCTCGAACCCTCCCGGCCCGCTCTCGCGTTGGCAGCATCACCCATAATTGTGGTTTCAAAATCGGCTCCGTCGATACTATGTTATACGCCAACTTTGAAAACAACTTTGAAAAAGCTGTTTTCTGGTATTTAAGGTTTTAGAATGCAAGGAACAGTGAATTGGAGTTCGTCTTGTTATAATTAGCTTCTTGGGGTATCTTTAAATACTGTAGAAAAGAGGAAGGAAATAATAAATGGCTAAAATGAGAATATCACCGGAATTGAAAAAACTGATCGAAAAATACCGCTGCGTAAAAGATACGGAAGGAATGTCTCCTGCTAAGGTATATAAGCTGGTGGGAGAAAATGAAAACCTATATTTAAAAATGACGGACAGCCGGTATAAAGGGACCACCTATGATGTGGAACGGGAAAAGGACATGATGCTATGGCTGGAAGGAAAGCTGCCTGTTCCAAAGGTCCTGCACTTTGAACGGCATGATGGCTGGAGCAATCTGCTCATGAGTGAGGCCGATGGCGTCCTTTGCTCGGAAGAGTATGAAGATGAACAAAGCCCTGAAAAGATTATCGAGCTGTATGCGGAGTGCATCAGGCTCTTTCACTCCATCGACATATCGGATTGTCCCTATACGAATAGCTTAGACAGCCGCTTAGCCGAATTGGATTACTTACTGAATAACGATCTGGCCGATGTGGATTGCGAAAACTGGGAAGAAGACACTCCATTTAAAGATCCGCGCGAGCTGTATGATTTTTTAAAGACGGAAAAGCCCGAAGAGGAACTTGTCTTTTCCCACGGCGACCTGGGAGACAGCAACATCTTTGTGAAAGATGGCAAAGTAAGTGGCTTTATTGATCTTGGGAGAAGCGGCAGGGCGGACAAGTGGTATGACATTGCCTTCTGCGTCCGGTCGATCAGGGAGGATATTGGGGAAGAACAGTATGTCGAGCTATTTTTTGACTTACTGGGGATCAAGCCTGATTGGGAGAAAATAAAATATTATATTTTACTGGATGAATTGTTTTAGTACCTAGATGTGGCGCAACGATGCCGGCGACAAGCAGGAGCGCACCGACTTCTTCCGCATCAAGTGTTTTGGCTCTCAGGCCGAGGCCCACGGCAAGTATTTGGGCAAGGGGTCGCTGGTATTCGTGCAGGGCAAGATTCGGAATACCAAGTACGAGAAGGACGGCCAGACGGTCTACGGGACCGACTTCATTGCCGATAAGGTGGATTATCTGGACACCAAGGCACCAGGCGGGTCAAATCAGGAATAAGGGCACATTGCCCCGGCGTGAGTCGGGGCAATCCCGCAAGGAGGGTGAATGAATCGGACGTTTGACCGGAAGGCATACAGGCAAGAACTGATCGACGCGGGGTTTTCCGCCGAGGATGCCGAAACCATCGCAAGCCGCACCGTCATGCGTGCGCCCCGCGAAACCTTCCAGTCCGTCGGCTCGATGGTCCAGCAAGCTACGGCCAAGATCGAGCGCGACAGCGTGCAACTGGCTCCCCCTGCCCTGCCCGCGCCATCGGCCGCCGTGGAGCGTTCGCGTCGTCTCGAACAGGAGGCGGCAGGTTTGGCGAAGTCGATGACCATCGACACGCGAGGAACTATGACGACCAAGAAGCGAAAAACCGCCGGCGAGGACCTGGCAAAACAGGTCAGCGAGGCCAAGCAAGCCGCGTTGCTGAAACACACGAAGCAGCAGATCAAGGAAATGCAGCTTTCCTTGTTCGATATTGCGCCGTGGCCGGACACGATGCGAGCGATGCCAAACGACACGGCCCGCTCTGCCCTGTTCACCACGCGCAACAAGAAAATCCCGCGCGAGGCGCTGCAAAACAAGGTCATTTTCCACGTCAACAAGGACGTGAAGATCACCTACACCGGCGTCGAGCTGCGGGCCGACGATGACGAACTGGTGTGGCAGCAGGTGTTGGAGTACGCGAAGCGCACCCCTATCGGCGAGCCGATCACCTTCACGTTCTACGAGCTTTGCCAGGACCTGGGCTGGTCGATCAATGGCCGGTATTACACGAAGGCCGAGGAATGCCTGTCGCGCCTACAGGCGACGGCGATGGGCTTCACGTCCGACCGCGTTGGGCACCTGGAATCGGTGTCGCTGCTGCACCGCTTCCGCGTCCTGGACCGTGGCAAGAAAACGTCCCGTTGCCAGGTCCTGATCGACGAGGAAATCGTCGTGCTGTTTGCTGGCGACCACTACACGAAATTCATATGGGAGAAGTACCGCAAGCTGTCGCCGACGGCCCGACGGATGTTCGACTATTTCAGCTCGCACCGGGAGCCGTACCCGCTCAAGCTGGAAACCTTCCGCCTCATGTGCGGATCGGATTCCACCCGCGTGAAGAAGTGGCGCGAGCAGGTCGGCGAAGCCTGCGAAGAGTTGCGAGGCAGCGGCCTGGTGGAACACGCCTGGGTCAATGATGACCTGGTGCATTGCAAACGCTAGGGCCTTGTGGGGTCAGTTCCGGCTGGGGGTTCAGCAGCCAGCGCTTTACTGAGATCCTCTTCCGCTTCCTCGCTCACTGACTCGCTGCGCTCGGTCGTTCGGCTGCGGCGAGCGGTATCAGCTCACTCAAAGGCGGTAATACGGTTATCCACAGAATCAGGGGATAACGCAGGAAAGAACATGTGAGCAAAAGGCCAGCAAAAGGCCAGGAACCGTAAAAAGGCCGCGTTGCTGGCGTTTTTCCATAGGCTCCGCCCCCCTGACGAGCATCACAAAAATCGACGCTCAAGTCAGAGGTGGCGAAACCCGACAGGACTATAAAGATACCAGGCGTTTCCCCCTGGAAGCTCCCTCGTGCGCTCTCCTGTTCCGACCCTGCCGCTTACCGGATACCTGTCCGCCTTTCTCCCTTCGGGAAGCGTGGCGCTTTCTCATAGCTCACGCTGTAGGTATCTCAGTTCGGTGTAGGTCGTTCGCTCCAAGCTGGGCTGTGTGCACGAACCCCCCGTTCAGCCCGACCGCTGCGCCTTATCCGGTAACTATCGTCTTGAGTCCAACCCGGTAAGACACGACTTATCGCCACTGGCAGCAGCCACTGGTAACAGGATTAGCAGAGCGAGGTATGTAGGCGGTGCTACAGAGTTCTTGAAGTGGTGGCCTAACTACGGCTACACTAGAAGAACAGTATTTGGTATCTGCGCTCTGCTGAAGCCAGTTACCTTCGGAAAAAGAGTTGGTAGCTCTTGATCCGGCAAACAAACCACCGCTGGTAGCGGTGGTTTTTTTGTTTGCAAGCAGCAGATTACGCGCAGAAAAAAAGGATCTCAAGAAGATCCTTTGATCTTTTCTACGGGGTCTGACGCTCAGTGGAACGAAAACTCACGTTAAGGGATTTTGGTCATGAGATTATCAAAAAGGATCTTCACCTAGATCCTTTTGGATCTCCTGTGGTTGGCATGCACATACAAATGGACGAACGGATAAACCTTTTCACGCCCTTTTAAATATCCGATTATTCTAATAAACGCTCTTTTCTCTTA

**pARS3-ZmUbi1-GUSPlus binary vector plasmid sequence**

**Annotation key: Right Border, nos terminator, GUSPlus, *catalase intron*, *Ubi1 intron*, Maize Ubi1 promoter, RUBQ2 promoter, *RUBQ2 intron***, **Ubiquitin monomer, hptII, 35S terminator, Left Border**

**GGTTTACCCGCCAATATATCCTGTCA**AACACTGATAGTTTCGCGTAGCGGATAACAATTTCACACAGGGATATCACTAGTAAAAGGTACCCCCGATCTAGTAACATAGATGACACCGCGCGCGATAATTTATCCTAGTTTGCGCGCTATATTTTGTTTTCTATCGCGTATTAAATGTATAATTGCGGGACTCTAATCATAAAAACCCATCTCATAAATAACGTCATGCATTACATGTTAATTATTACATGCTTAACGTAATTCAACAGAAATTATATGATAATCATCGCAAGACCGGCAACAGGATTCAATCTTAAGAAACTTTATTGCCAAATGTTTGAACGATCGGGGAAATTCGAGCTGGTCACCAATTCACACGTGATGGTGATGGTGATGGCTAGCGTTCTTGTAGCCGAAATCTGGAATGTTGGTCCAGCGCTCGCGAAAGACGTGCGCGGCGAGCTTCGGCTTGCGGTCACGAGTGAACACGCCCTTCTTGTTTCCTTGGACGCGCATCACGCCCTGAGAGGTCGCGAAGTCCGCGAAGTTCCACGCTTGCTCACCCACGAAGTTCTCAAACTCATCGAACACGACGTGGTTCGCCTGGTAGTACTCGACTTGATATTCCTCGGTGAACATCACTGGATCAATGTCGTGAAAGCCCGCAACGGTGTCTGCGCCGTACTCAGTGATCATGATCGGCTTTCCTGGGCAACGCTTGTTCCACGCGTGAAATTCCTGGCGGAGATGGACTTTGGCCGCTTCGAGATCACCGCCATCGAAGTACCATCCGTTATAGCGATTGAGCGCGATGACGTCAATCAGTTCGGCGACTTTGTCCGTCTCCGGGGTAGCCATCACAAACAGCACGATCGTGACCGGACGCTTCTGTGGGTCGAGTTCCTTGGTCAGCTCCACCAACGGCTTGAAGTACTCGTACGCGCCCTCTTCCTCAGTCGCCGCCTCGTTGGCGATGCTCCACATCACGACGCTTGGATGGTTCTTGTCACGAGACACCAGTTCACGGAGAACGTCTTGATGGTGCTCAAACGTCCGAATCTTCTCCCAGGTACTGACGCGCTCGCTGCCTTCGCCGAGTCCCGTGGTGGCCATGAAGTTGAGGTGCACGCCAACTGCCGGAGTCTCGTCGATCACGACCAGACCCTCGCGATCCGCAAGACGCATCAACTCTTCAGAGTACGGATAGTGTGCGGTCCGGAAGCTGTTGGCGCCGATCCATTTGAGGATATTGAAATCCATCACATTGCTCGCTTCGTTAAAGCCACGGCCGTTGATAGGAGTGTCCTCATGTTTGCCAAAGCCCTTGAAGTAGAACGGTTTGTTGTTGATGAGGAACTTGCCGTCGTTGACTTCCACGGTCCGCACGCCGAACGGCTCTTCATAGACATCGATGGTCAGTCCGTCGTTCACCAGTTCCACTTTGATCTGGTAGAGATACGTGTTCAGTGGTTCCCAGAGGATGACATTCGGAATCTCCACGTTACCGCTCAGGCCCTCGGTGCTTGCGACCACTTTGCCTTCCTCATCCACGACCGACACTTTCACGGTCTCGGCTTTGCCTTGAAAGTCCACCGTATAGGTCACAGTCCCGGTTGGGCCATTGAAGTCGGTCACAACCGAGATGTCCTCGACGTACGTAAACGGGGTCGTGTAGATTTTCACCGGACGGTGCAGGCCTGCATAGTTGAAGAAGTCGAAGTTCGGCTTGTTACGAATGACTTTTCCGAGGCCCTCTTCGTGGCGCTCGCTGTACAGCCCCACCGGGAGGGTGCTATCGTCGAGGATGTTGTCCACGGCGACGGTGACGCGATTCATGCCATCACGCAGCGAGTTGTTGATTTCCGCTTCGAATGGCAGGAATCCGCCCTTGTGCTCCACGACCAGCTCACCATTGACATAGACAATTGCTTTGTGAGTTGCAGAGCCGAAGCGGAGCACGATACGCTGATCCTTCAGATAGGCCGGCACCGTGAACTCACGTTCGTACCAGACATATCCGATATGGTTGCGGATTTCCTTGGTCACGCCAATGTCATTGTAACTGCTTGGGACGGCCATACTAATAGTGTCGGTCAGCTTGCTTTCGTACCACTTCTCTTCCAGTCCTTTCCCGTAGTCCAGCTTGAAGTTCCAGACGCCATTGAGGTCGAAGACGCCACGGGTCTCGGTGTTGATCGGGTACAGACTAGTTCGTCGGTT*CTGTAACTATCATCATCATCATAGACACACGAAATAAAGTAATCAGATTATCAGTTAAAGCTATGTAATATTTACACCATAACCAATCAATTAAAAAATAGATCAGTTTAAAGAAAGATCAAAGCTCAAAAAAATAAAAAGAGAAAAGGGTCCTAACCAAGAAAATGAAGGAGAAAAACTAGAAATTTAC*CCTCAGATCTACCATGGGGTACCAGTTTGATCCTCTAGAGTCGAC***CTGCAGAAGTAACACCAAACAACAGGGTGAGCATCGACAAAAGAAACAGTACCAAGCAAATAAATAGCGTATGAAGGCAGGGCTAAAAAAATCCACATATAGCTGCTGCATATGCCATCATCCAAGTATATCAAGATCAAAATAATTATAAAACATACTTGTTTATTATAATAGATAGGTACTCAAGGTTAGAGCATATGAATAGATGCTGCATATGCCATCATGTATATGCATCAGTAAAACCCACATCAACATGTATACCTATCCTAGATCGATATTTCCATCCATCTTAAACTCGTAACTATGAAGATGTATGACACACACATACAGTTCCAAAATTAATAAATACACCAGGTAGTTTGAAACAGTATTCTACTCCGATCTAGAACGAATGAACGACCGCCCAACCACACCACATCATCACAACCAAGCGAACAAAAAGCATCTCTGTATATGCATCAGTAAAACCCGCATCAACATGTATACCTATCCTAGATCGATATTTCCATCCATCATCTTCAATTCGTAACTATGAATATGTATGGCACACACATACAGATCCAAAATTAATAAATCCACCAGGTAGTTTGAAACAGATTTCTACTCCGATCTAGAACGACCGCCCAACCAGACCACATCATCACAACCAAGACAAAAAAAAGCATGAAAAGATGACCCGACAAACAAGTGCACGGCATATATTGAAATAAAGGAAAAGGGCAAACCAAACCCTATGCAACGAAACAAAAAAAATCATGAAATCGATCCCGTCTGCGGAACGGCTAGAGCCATCCCAGGATTCCCCAAAGAGAAACACTGGCAAGTTAGCAATCAGAACGTGTCTGACGTACAGGTCGCATCCGTGTACGAACGCTAGCAGCACGGATCTAACACAAACACGGATCTAACACAAACATGAACAGAAGTAGAACTACCGGGCCCTAACCATGGACCGGAACGCCGATCTAGAGAAGGTAGAGAGGGGGGGGGGGGGAGGAC***GAGCGGCGTACCTTGAAGCGGAGGTGCCGACGGGTGGATTTGGGGGAGATCTGGTTGTGTGTGTGTGCGCTCCGAACAACACGAGGTTGGGGAAAGAGGGTGTGGAGGGGGTGTCTATTTATTACGGCGGGCGAGGAAGGGAAAGCGAAGGAGCGGTGGGAAAGGAATCCCCCGTAGCTGCCGGTGCCGTGAGAGGAGGAGGAGGCCGCCTGCCGTGCCGGCTCACGTCTGCCGCTCCGCCACGCAATTTCTGGATGCCGACAGCGGAGCAAGTCCAACGGTGGAGCGGAACTCTCGAGAGGGGTCCAGAGGCAGCGACAGAGATGCCGTGCCGTCTGCTTCGCTTGGCCCGACGCGACGCTGCTGGTTCGCTGGTTGGTGTCCGTTAGACTCGTCGACGGCGTTTAACAGGCTGGCATTATCTACTCGAAACAAGAAAAATGTTTCCTTAGTTTTTTTAATTTCTTAAAGGGTATTTGTTTAATTTTTAGTCACTTTATTTTATTCTATTTTATATCTAAATTATTAAATAAAAAAACTAAAATAGAGTTTTAGTTTTCTTAATTTAGAGGCTAAAATAGAATAAAATAGATGTACTAAAAAAATTAGTCTATAAAAACCATTAACCCTAAACCCTAAATGGATGTACTAATAAAATGGATGAAGTATTATATAGGTGAAGCTATTTGCAAAAAAAAAGGAGAACACATGCACACTAAAAAGATAAAACTGTAGAGTCCTGTTGTCAAAATACTCAATTGTCCTTTAGACCATGTCTAACTGTTCATTTATATGATTCTCTAAAACACTGATATTATTGTAGTACTATAGATTATATTATTCGTAGAGTAAAGTTTAAATATATGTATAAAGATAGATAAACTGCACTTCAAACAAGTGTGACAAAAAAAATATGTGGTAATTTTTTATAACTTAGACATGCAATGCTCATTATCTCTAGAGAGGGGCACGACCGGGTCACGCTGCACTGCAGGCATGCAAGCTTATTCGGGTCAAGGGGAAGCCAGCGCGCCACCCCACGTCAGCAAATACGGAGGCGCGGGGTTGACGGCGTCACCCGGTCCTAACGGCGACCAACAAACCAGCCAGAAGAAATTACAGTAAAAAAAAAGTAAATTGCACTTTGATCCACCTTTTATTACCTAAGTCTCAATTTGGATCACCCTTAAACCTATCTTTTCAATTTGGGCCGGGTTGTGGTTTGGACTACCATGAACAACTTTTCGTCATGTCTAACTTCCCTTTCAGCAAACATATGAACCATATATAGAGGAGATCGGCCGTATACTAGAGCTGATGTGTTTAAGGTCGTTGATTGCACGAGAAAAAAAAATCCAAATCGCAACAATAGCAAATTTATCTGGTTCAAAGTGAAAAGATATGTTTAAAGGTAGTCCAAAGTAAAACTTATAGATAATAAAATGTGGTCCAAAGCGTAATTCACTCAAAAAAAATCAACGAGACGTGTACCAAACGGAGACAAACGGCATCTTCTCGAAATTTCCCAACCGCTCGCTCGCCCGCCTCGTCTTCCCGGAAACCGCGGTGGTTTCAGCGTGGCGGATTCTCCAAGCAGACGGAGACGTCACGGCACGGGACTCCTCCCACCACCCAACCGCCATAAATACCAGCCCCCTCATCTCCTCTCCTCGCATCAGCTCCACCCCCGAAAAATTTCTCCCCAATCTCGCGAGGCTCTCGTCGTCGAATCGAATCCTCTCGCGTCCTCAAG***GTACGCTGCTTCTCCTCTCCTCGCTTCGTTTCGATTCGATTTCGGACGGGTGAGGTTGTTTTGTTGCTAGATCCGATTGGTGGTTAGGGTTGTCGATGTGATTATCGTGAGATGTTTAGGGGTTGTAGATCTGATGGTTGTGATTTGGGCACGGTTGGTTCGATAGGTGGAATCGTGGTTAGGTTTTGGGATTGGATGTTGGTTCTGATGATTGGGGGGAATTTTTACGGTTAGATGAATTGTTGGATGATTCGATTGGGGAAATCGGTGTAGATCTGTTGGGGAATTGTGGAACTAGTCATGCCTGAGTGATTGGTGCGATTTGTAGCGTGTTCCATCTTGTAGGCCTTGTTGCGAGCATGTTCAGATCTACTGTTCCGCTCTTGATTGAGTTATTGGTGCCTTGGGTTGGTGCAAACACAGGCTTTAATATGTTATATCTGTTTTGTGTTTGATGTAGATCTGTAGGGTAGTTCTTCTTAGACATGGTTCAATTATGTAGCTTGTGCGTTTCGATTTGATTTCATATGTTCACAGATTAGATAATGATGAACTCTTTTAATTAATTGTCAATGGTAAATAGGAAGTCTTGTCGCTATATCTGTCATAATGATCTCATGTTACTATCTGCCAGTAATTTATGCTAAGAACTATATTAGAATATCATGTTACAATCTGTAGTAATATCATGTTACAATCTGTAGTTCATCTATATAATCTATTGTGGTAATTTCTTTTTACTATCTGTGTGAAGATTATTGCCACTAGTTCATTCTACTTATTTCTGAAGTTCAGGATACGTGTGCTGTTACTACCTATCTGAATACATGTGTGATGTGCCTGTTACTATCTTTTTGAATACATGTATGTTCTGTTGGAATATGTTTGCTGTTTGATCCGTTGTTGTGTCCTTAATCTTGTGCTAGTTCTTACCCTATCTGTTTGGTGATTATTTCTTGCAG***ATGCAGATCTTTGTGAAGACATTGACCGGCAAGACTATCACCCTGGAGGTGGAGTCCTCTGACACCATCGACAATGTCAAGGCTAAGATCCAAGATAAGGAGGGCATCCCCCCGGACCAGCAGCGTCTCATCTTCGCTGGCAAGCAGCTCGAAGATGGCAGGACCCTTGCTGACTACAACATCCAGAAGGAGTCCACCCTTCACCTTGTCCTCCGCCTCCGTGGTGGCGGATCCATGAAAAAGCCTGAACTCACCGCGACGTCTGTCGAGAAGTTTCTGATCGAAAAGTTCGACAGCGTCTCCGACCTGATGCAGCTCTCGGAGGGCGAAGAATCTCGTGCTTTCAGCTTCGATGTAGGAGGGCGTGGATATGTCCTGCGGGTAAATAGCTGCGCCGATGGTTTCTACAAAGATCGTTATGTTTATCGGCACTTTGCATCGGCCGCGCTCCCGATTCCGGAAGTGCTTGACATTGGGGAGTTTAGCGAGAGCCTGACCTATTGCATCTCCCGCCGTTCACAGGGTGTCACGTTGCAAGACCTGCCTGAAACCGAACTGCCCGCTGTTCTACAACCGGTCGCGGAGGCTATGGATGCGATCGCTGCGGCCGATCTTAGCCAGACGAGCGGGTTCGGCCCATTCGGACCGCAAGGAATCGGTCAATACACTACATGGCGTGATTTCATATGCGCGATTGCTGATCCCCATGTGTATCACTGGCAAACTGTGATGGACGACACCGTCAGTGCGTCCGTCGCGCAGGCTCTCGATGAGCTGATGCTTTGGGCCGAGGACTGCCCCGAAGTCCGGCACCTCGTGCACGCGGATTTCGGCTCCAACAATGTCCTGACGGACAATGGCCGCATAACAGCGGTCATTGACTGGAGCGAGGCGATGTTCGGGGATTCCCAATACGAGGTCGCCAACATCTTCTTCTGGAGGCCGTGGTTGGCTTGTATGGAGCAGCAGACGCGCTACTTCGAGCGGAGGCATCCGGAGCTTGCAGGATCGCCACGACTCCGGGCGTATATGCTCCGCATTGGTCTTGACCAACTCTATCAGAGCTTGGTTGACGGCAATTTCGATGATGCAGCTTGGGCGCAGGGTCGATGCGACGCAATCGTCCGATCCGGAGCCGGGACTGTCGGGCGTACACAAATCGCCCGCAGAAGCGCGGCCGTCTGGACCGATGGCTGTGTAGAAGTACTCGCCGATAGTGGAAACCGACGCCCCAGCACTCGTCCGAGGGCAAAGAAATAGTTAATCGACAAGCTCGAGTTTCTCCATAATAATGTGTGAGTAGTTCCCAGATAAGGGAATTAGGGTTCCTATAGGGTTTCGCTCATGTGTTGAGCATATAAGAAACCCTTAGTATGTATTTGTATTTGTAAAATACTTCTATCAATAAAATTTCTAATTCCTAAAACCAAAATCCAGTACTAAAATCCAGATCCCCCGAATTAATTCGGCGTTAATTCAGTACATTAAAAACGCGTACGGTTAAAACCACCCCAGTACATTAAAAACGTCCGCAATGTGTTATTAAGTTGTCTAAGCGTCAATTTGTTTACACCACAATATATCCTGCCACCAGCCAGCCAACAGCTCCCCGACCGGCAGCTCGGCACAAAATCACCACTCGATACAGGCAGCCCATCAGTCCACTAGACGCTCACCGGGCTGGTTGCCCTCGCCGCTGGGCTGGCGGCCGTCTATGGCCCTGCAAACGCGCCAGAAACGCCGTCGAAGCCGTGTGCGAGACACCGCAGCCGCCGGCGTTGTGGATACCTCGCGGAAAACTTGGCCCTCACTGACAGATGAGGGGCGGACGTTGACACTTGAGGGGCCGACTCACCCGGCGCGGCGTTGACAGATGAGGGGCAGGCTCGATTTCGGCCGGCGACGTGGAGCTGGCCAGCCTCGCAAATCGGCGAAAACGCCTGATTTTACGCGAGTTTCCCACAGATGATGTGGACAAGCCTGGGGATAAGTGCCCTGCGGTATTGACACTTGAGGGGCGCGACTACTGACAGATGAGGGGCGCGATCCTTGACACTTGAGGGGCAGAGTGCTGACAGATGAGGGGCGCACCTATTGACATTTGAGGGGCTGTCCACAGGCAGAAAATCCAGCATTTGCAAGGGTTTCCGCCCGTTTTTCGGCCACCGCTAACCTGTCTTTTAACCTGCTTTTAAACCAATATTTATAAACCTTGTTTTTAACCAGGGCTGCGCCCTGTGCGCGTGACCGCGCACGCCGAAGGGGGGTGCCCCCCCTTCTCGAACCCTCCCGGCCCGCTCTCGCGTTGGCAGCATCACCCATAATTGTGGTTTCAAAATCGGCTCCGTCGATACTATGTTATACGCCAACTTTGAAAACAACTTTGAAAAAGCTGTTTTCTGGTATTTAAGGTTTTAGAATGCAAGGAACAGTGAATTGGAGTTCGTCTTGTTATAATTAGCTTCTTGGGGTATCTTTAAATACTGTAGAAAAGAGGAAGGAAATAATAAATGGCTAAAATGAGAATATCACCGGAATTGAAAAAACTGATCGAAAAATACCGCTGCGTAAAAGATACGGAAGGAATGTCTCCTGCTAAGGTATATAAGCTGGTGGGAGAAAATGAAAACCTATATTTAAAAATGACGGACAGCCGGTATAAAGGGACCACCTATGATGTGGAACGGGAAAAGGACATGATGCTATGGCTGGAAGGAAAGCTGCCTGTTCCAAAGGTCCTGCACTTTGAACGGCATGATGGCTGGAGCAATCTGCTCATGAGTGAGGCCGATGGCGTCCTTTGCTCGGAAGAGTATGAAGATGAACAAAGCCCTGAAAAGATTATCGAGCTGTATGCGGAGTGCATCAGGCTCTTTCACTCCATCGACATATCGGATTGTCCCTATACGAATAGCTTAGACAGCCGCTTAGCCGAATTGGATTACTTACTGAATAACGATCTGGCCGATGTGGATTGCGAAAACTGGGAAGAAGACACTCCATTTAAAGATCCGCGCGAGCTGTATGATTTTTTAAAGACGGAAAAGCCCGAAGAGGAACTTGTCTTTTCCCACGGCGACCTGGGAGACAGCAACATCTTTGTGAAAGATGGCAAAGTAAGTGGCTTTATTGATCTTGGGAGAAGCGGCAGGGCGGACAAGTGGTATGACATTGCCTTCTGCGTCCGGTCGATCAGGGAGGATATTGGGGAAGAACAGTATGTCGAGCTATTTTTTGACTTACTGGGGATCAAGCCTGATTGGGAGAAAATAAAATATTATATTTTACTGGATGAATTGTTTTAGTACCTAGATGTGGCGCAACGATGCCGGCGACAAGCAGGAGCGCACCGACTTCTTCCGCATCAAGTGTTTTGGCTCTCAGGCCGAGGCCCACGGCAAGTATTTGGGCAAGGGGTCGCTGGTATTCGTGCAGGGCAAGATTCGGAATACCAAGTACGAGAAGGACGGCCAGACGGTCTACGGGACCGACTTCATTGCCGATAAGGTGGATTATCTGGACACCAAGGCACCAGGCGGGTCAAATCAGGAATAAGGGCACATTGCCCCGGCGTGAGTCGGGGCAATCCCGCAAGGAGGGTGAATGAATCGGACGTTTGACCGGAAGGCATACAGGCAAGAACTGATCGACGCGGGGTTTTCCGCCGAGGATGCCGAAACCATCGCAAGCCGCACCGTCATGCGTGCGCCCCGCGAAACCTTCCAGTCCGTCGGCTCGATGGTCCAGCAAGCTACGGCCAAGATCGAGCGCGACAGCGTGCAACTGGCTCCCCCTGCCCTGCCCGCGCCATCGGCCGCCGTGGAGCGTTCGCGTCGTCTCGAACAGGAGGCGGCAGGTTTGGCGAAGTCGATGACCATCGACACGCGAGGAACTATGACGACCAAGAAGCGAAAAACCGCCGGCGAGGACCTGGCAAAACAGGTCAGCGAGGCCAAGCAAGCCGCGTTGCTGAAACACACGAAGCAGCAGATCAAGGAAATGCAGCTTTCCTTGTTCGATATTGCGCCGTGGCCGGACACGATGCGAGCGATGCCAAACGACACGGCCCGCTCTGCCCTGTTCACCACGCGCAACAAGAAAATCCCGCGCGAGGCGCTGCAAAACAAGGTCATTTTCCACGTCAACAAGGACGTGAAGATCACCTACACCGGCGTCGAGCTGCGGGCCGACGATGACGAACTGGTGTGGCAGCAGGTGTTGGAGTACGCGAAGCGCACCCCTATCGGCGAGCCGATCACCTTCACGTTCTACGAGCTTTGCCAGGACCTGGGCTGGTCGATCAATGGCCGGTATTACACGAAGGCCGAGGAATGCCTGTCGCGCCTACAGGCGACGGCGATGGGCTTCACGTCCGACCGCGTTGGGCACCTGGAATCGGTGTCGCTGCTGCACCGCTTCCGCGTCCTGGACCGTGGCAAGAAAACGTCCCGTTGCCAGGTCCTGATCGACGAGGAAATCGTCGTGCTGTTTGCTGGCGACCACTACACGAAATTCATATGGGAGAAGTACCGCAAGCTGTCGCCGACGGCCCGACGGATGTTCGACTATTTCAGCTCGCACCGGGAGCCGTACCCGCTCAAGCTGGAAACCTTCCGCCTCATGTGCGGATCGGATTCCACCCGCGTGAAGAAGTGGCGCGAGCAGGTCGGCGAAGCCTGCGAAGAGTTGCGAGGCAGCGGCCTGGTGGAACACGCCTGGGTCAATGATGACCTGGTGCATTGCAAACGCTAGGGCCTTGTGGGGTCAGTTCCGGCTGGGGGTTCAGCAGCCAGCGCTTTACTGAGATCCTCTTCCGCTTCCTCGCTCACTGACTCGCTGCGCTCGGTCGTTCGGCTGCGGCGAGCGGTATCAGCTCACTCAAAGGCGGTAATACGGTTATCCACAGAATCAGGGGATAACGCAGGAAAGAACATGTGAGCAAAAGGCCAGCAAAAGGCCAGGAACCGTAAAAAGGCCGCGTTGCTGGCGTTTTTCCATAGGCTCCGCCCCCCTGACGAGCATCACAAAAATCGACGCTCAAGTCAGAGGTGGCGAAACCCGACAGGACTATAAAGATACCAGGCGTTTCCCCCTGGAAGCTCCCTCGTGCGCTCTCCTGTTCCGACCCTGCCGCTTACCGGATACCTGTCCGCCTTTCTCCCTTCGGGAAGCGTGGCGCTTTCTCATAGCTCACGCTGTAGGTATCTCAGTTCGGTGTAGGTCGTTCGCTCCAAGCTGGGCTGTGTGCACGAACCCCCCGTTCAGCCCGACCGCTGCGCCTTATCCGGTAACTATCGTCTTGAGTCCAACCCGGTAAGACACGACTTATCGCCACTGGCAGCAGCCACTGGTAACAGGATTAGCAGAGCGAGGTATGTAGGCGGTGCTACAGAGTTCTTGAAGTGGTGGCCTAACTACGGCTACACTAGAAGAACAGTATTTGGTATCTGCGCTCTGCTGAAGCCAGTTACCTTCGGAAAAAGAGTTGGTAGCTCTTGATCCGGCAAACAAACCACCGCTGGTAGCGGTGGTTTTTTTGTTTGCAAGCAGCAGATTACGCGCAGAAAAAAAGGATCTCAAGAAGATCCTTTGATCTTTTCTACGGGGTCTGACGCTCAGTGGAACGAAAACTCACGTTAAGGGATTTTGGTCATGAGATTATCAAAAAGGATCTTCACCTAGATCCTTTTGGATCTCCTGTGGTTGGCATGCACATACAAATGGACGAACGGATAAACCTTTTCACGCCCTTTTAAATATCCGATTATTCTAATAAACGCTCTTTTCTCTTA
