# Supplementary material for: Use of Agrobacterium rhizogenes Strain 18r12v and Paromomycin Selection for Transformation of Brachypodium distachyon and Brachypodium sylvaticum
Source: Front Plant Sci. 2016 May 24;7:716. doi: 10.3389/fpls.2016.00716 (PMC4877385; doi:10.3389/fpls.2016.00716)
Supplement: Supplementary file 2 [file Data_Sheet_2.DOCX]

**
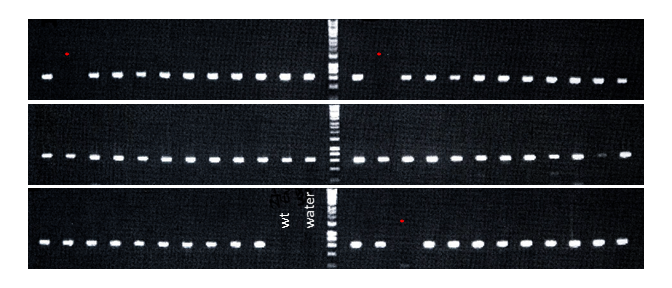
**

**Supplemental File 2. Genotyping of transgenic T_0_ *B. distachyon*.** Leaf samples were tested for the presence of the *nptII* gene by genomic PCR. These gel images show a representative set of PCR results for 70 tested regenerant T_0_ plants. A wild-type (wt) Bd21-3 leaf sample and a water blank were included as negative controls. Bands indicating the presence of the *nptII* gene are seen for 67 of the 70 plants tested in this set (96%). A band fails to amplify in three of the samples (red asterisks, 4%), suggesting that these plants are nontransgenic escapes. Samples were loaded into every other lane of the 1% agarose gel using a multichannel pipet. The size marker in the center lane is Hyperladder 1 kb.
